# Supplementary material for: An exposome atlas of serum reveals the risk of chronic diseases in the Chinese population
Source: Nat Commun. 2024 Mar 13;15:2268. doi: 10.1038/s41467-024-46595-z (PMC10937660; doi:10.1038/s41467-024-46595-z)
Supplement: Supplementary file 1 — Supplementary information [file 41467_2024_46595_MOESM1_ESM.pdf]

Supplementary information for

An exposome atlas of serum reveals the risk of chronic diseases in the Chinese population

Lei You[1,2,3,8], Jing Kou[4,8], Mengdie Wang[1,3,5,8], Guoqin Ji[1,3,6,8], Xiang Li[4], Chang Su[7], Fujian Zheng[1,2,3], Mingye Zhang[4], Yuting Wang[1,2,3], Tiantian Chen[1,2,3], Ting Li[1,2,3], Lina Zhou[1,2,3], Xianzhe Shi[1,2,3], Chunxia Zhao[1,2,3], Xinyu Liu[1,2,3*], Surong Mei[4*], Guowang Xu[1,2,3*]

1. CAS Key Laboratory of Separation Science for Analytical Chemistry, Dalian Institute of Chemical Physics, Chinese Academy of Sciences, 457 Zhongshan Road, Dalian 116023, China
2. University of Chinese Academy of Sciences, Beijing 100049, China
3. Liaoning Province Key Laboratory of Metabolomics, Dalian 116023, China
4. State Key Laboratory of Environment Health (Incubation), Key Laboratory of Environment and Health, Ministry of Education, Key Laboratory of Environment and Health (Wuhan), Ministry of Environmental Protection, School of Public Health, Tongji Medical College, Huazhong University of Science and Technology, # 13 Hangkong Road, Wuhan, Hubei, 430030, China
5. School of Public Health, China Medical University, No. 77 Puhe Road, Shenbei New District, Shenyang, 110122, China
6. School of life science, China Medical University, No. 77 Puhe Road, Shenbei New District, Shenyang, 110122, China
7. National Institute for Nutrition and Health, Chinese Center for Disease Control and Prevention, Beijing 100050, China
8. These authors contributed equally: Lei You, Jing Kou, Mengdie Wang, Guoqin Ji

e-mail addresses:

*: Corresponding authors:

Xinyu Liu, liuxy2012@dicp.ac.cn

Surong Mei, surongmei@hust.edu.cn

Guowang Xu, xugw@dicp.ac.cn

List of Supplementary Figures

Supplementary Figure 1: The classification of detected exposures.

Supplementary Figure 2. Running sequence of calibration curves, quality control samples and real samples in instrumental analysis.

Supplementary Figure 3: Accuracy of each calibration curves at low, medium, and high spiked concentrations.

Supplementary Figure 4: Batch effect correction in LC-MS/MS platform.

Supplementary Figure 5: The evaluation of data quality by QC samples.

Supplementary Figure 6: Sample information of basic epidemiological factors.

Supplementary Figure 7: Principal component analysis of basic epidemiology factors.

Supplementary Figure 8: Correlations among high-frequency exposures.

Supplementary Figure 9: Regional distribution of different categories of exposures.

Supplementary Figure 10: The plasma/serum exposures profile in different countries.

Supplementary Figure 11: Exposure levels in different populations.

Supplementary Figure 12: Sample information of chronic diseases.

Supplementary Figure 13: The risk of exposures to chronic diseases.

Supplementary Figure 14: The odds ratios of exposure to chronic diseases after considering medical history and external environment factors.

Supplementary Figure 15: The odds ratios of all exposures to chronic diseases (part 1).

Supplementary Figure 16: The odds ratios of all exposures to chronic diseases (part 2).

Supplementary Figure 17: Correlation network of high-frequency exposures and 9 clinical disease parameters.

Supplementary Figure 18: Risk analysis stratified by gender.

Supplementary Figure 19: Health risk assessment and the determination of exposure guidance values.

Supplementary Figure 20: The risk of exposure mixtures to chronic diseases.

Supplementary Figure 21: Dose-risk relationship between key exposures and related chronic disease.

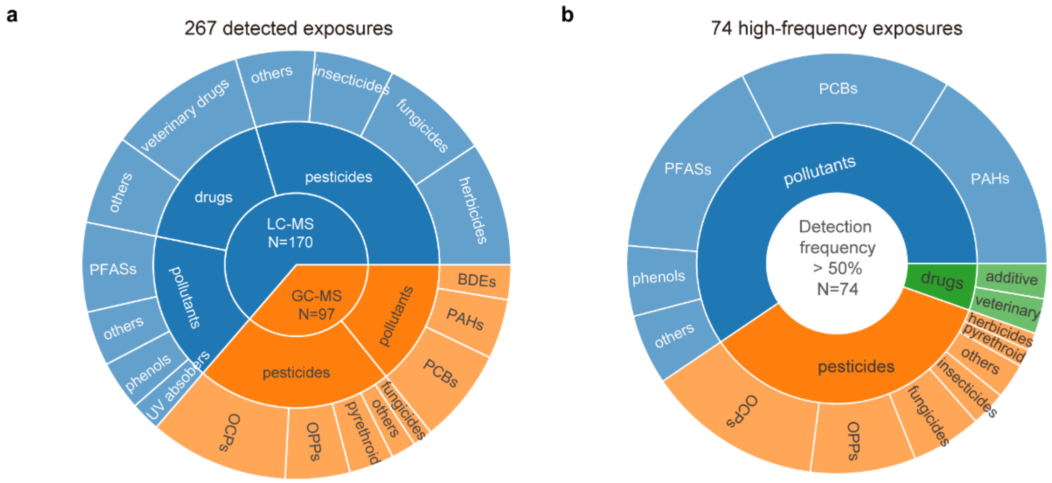

Supplementary Figure 1. The classification of detected exposures. a 267 exposures were detected by two platforms of LC-MS/MS and GC-MS/MS. **b** 74 high-frequency exposures are determined based on the detection rate above 50% in any one of 15 provinces. OCP, organochlorine pesticide; OPP, organophosphorus pesticide; PAH, polycyclic aromatic hydrocarbon; PCB, polychlorinated biphenyl; PFAS, perfluoroalkyl substance.

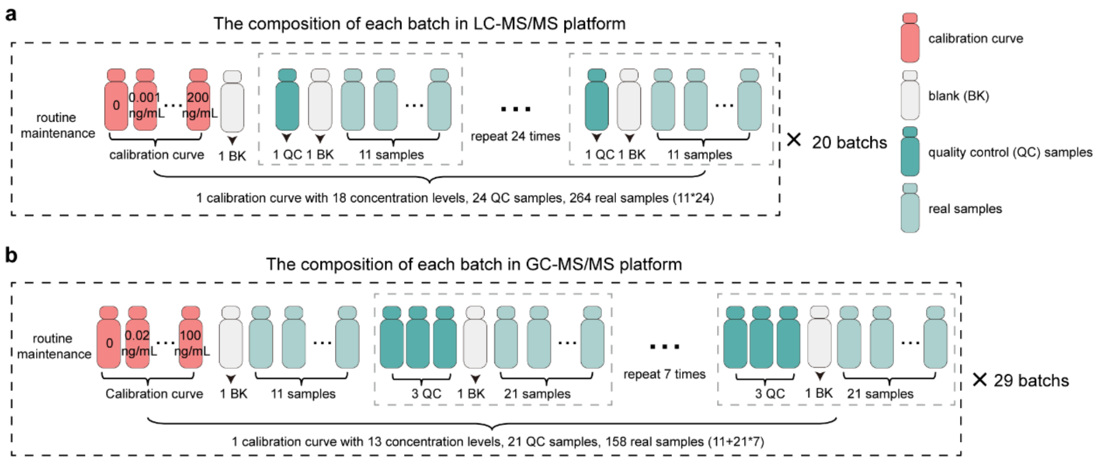

Supplementary Figure 2. Running sequence of calibration curves, quality control samples and real samples in instrumental analysis. a The composition of each batch in LC-MS/MS platform. **b** The composition of each batch in GC-MS/MS platform.

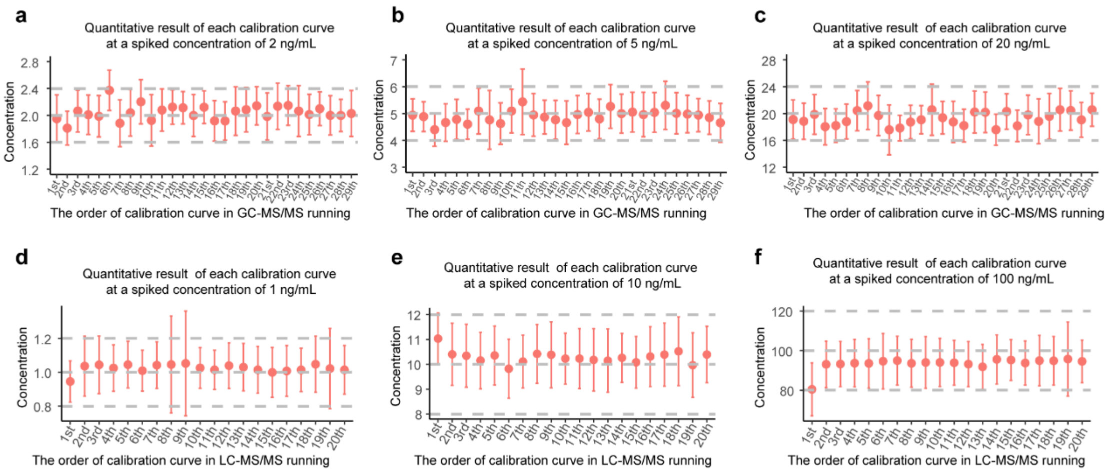

Supplementary Figure 3. Accuracy of each calibration curves at low, medium, and high spiked concentrations. a-c Quantitative concentrations of 29 calibration curves at 2, 5, and 20 ng/mL spiked concentrations in GC-MS/MS platform. **d-f** Quantitative concentrations of 20 calibration curves at 1, 10, and 100 ng/mL spiked concentrations in LC-MS/MS platform. Points represent mean concentrations of all detected chemicals and error bars represent standard deviation of mean. Each dashed line in middle of plot represents the actual spiked concentration, while the upper and lower dashed lines represent the quantification concentrations at 80% and 120% accuracy, respectively.

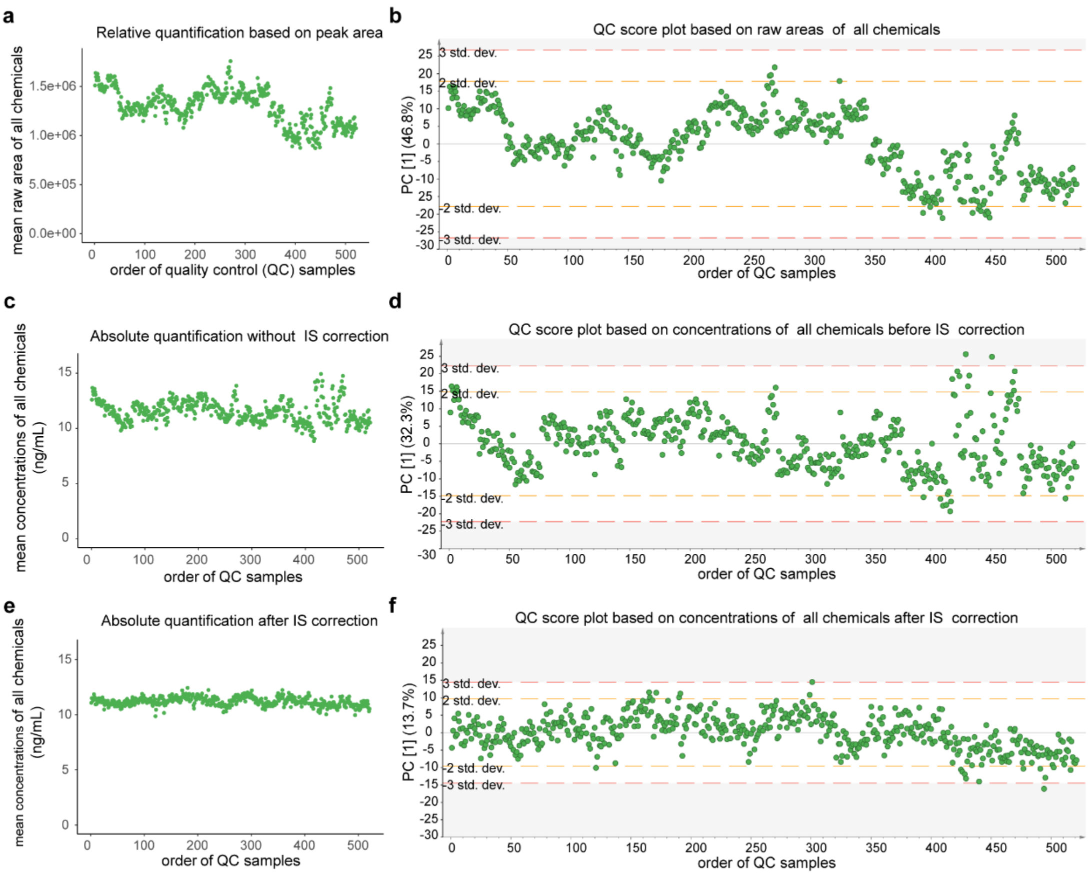

Supplementary Figure 4. Batch effect correction in LC-MS/MS platform. a Mean raw area of all detected chemicals in 521 QC samples. **b** QC score plot of principal component analysis based on the raw areas of all chemicals. **c** Mean absolute concentration of all chemicals in all QC samples before internal standard (IS) correction. **d** QC score plot of principal component analysis based on the absolute concentration of all chemicals before IS correction. **e** Mean absolute concentration of all chemicals in all QC samples after IS correction. **f** QC score plot of principal component analysis based on the absolute concentration of all detected chemicals after IS correction.

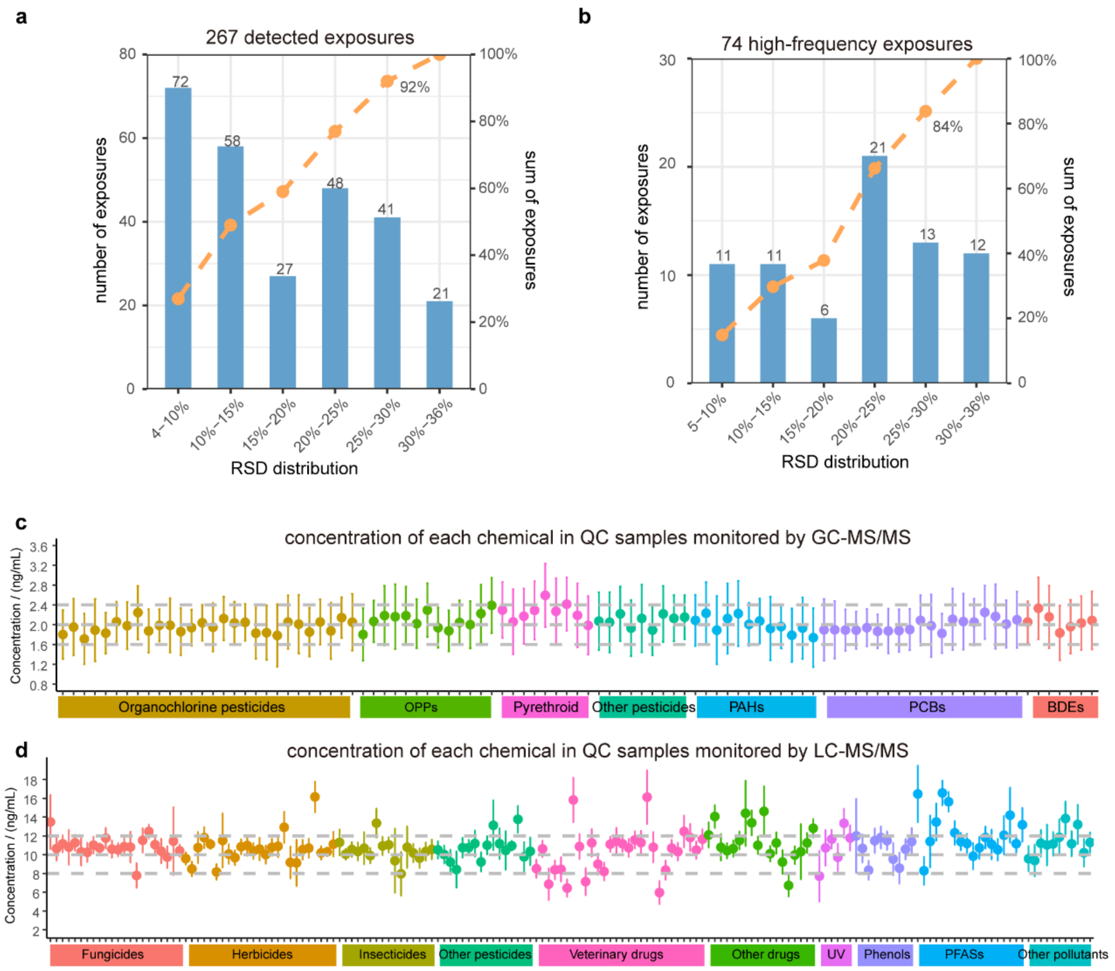

Supplementary Figure 5. The evaluation of data quality by QC samples. a-b Relative standard deviation (RSD) distribution of all exposures and high-frequency exposures in QC samples for evaluating method repeatability. **c-d** The quantified concentration of QC samples based on GC-MS/MS and LC-MS/MS platform, respectively. Points represent mean concentration of each detected chemical and error bars represent standard deviation of mean. Each dashed line in middle of figure c and d represents the actual spiked concentration, while the upper and lower dashed lines represent the quantification concentrations at 80% and 120% accuracy, respectively.

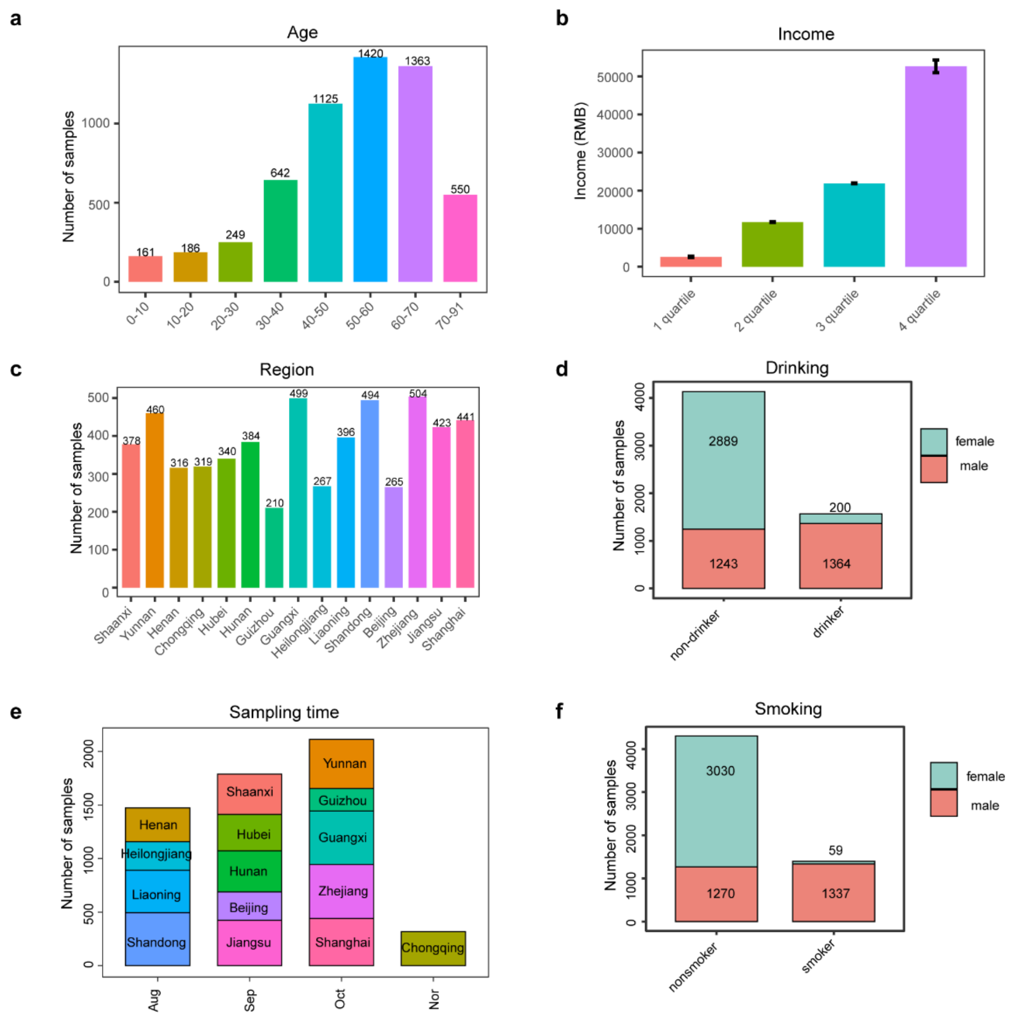

Supplementary Figure 6. Sample information of basic epidemiological factors. a Number of samples in different age ranges. **b** The income levels of each quartile. **c** Number of samples in each of 15 provinces. **d** Number of samples of non-drinker and drinker after the gender layer. **e** Number of samples in different sampling time and their relationship with the sampling provinces. **f** Number of samples of non-smoker and smoker after the gender layer.

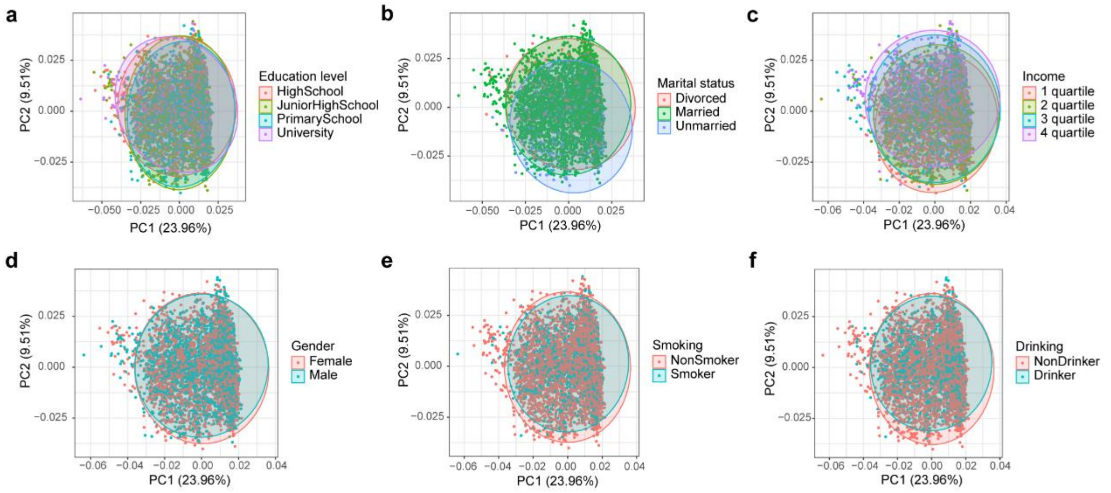

Supplementary Figure 7. Principal component analysis of basic epidemiology factors. The distinction among 4573 samples by using different education levels (**a**), marital status (**b**), income levels (**c**), gender (**d**), smoking history (**e**), and drinking history (**f**).

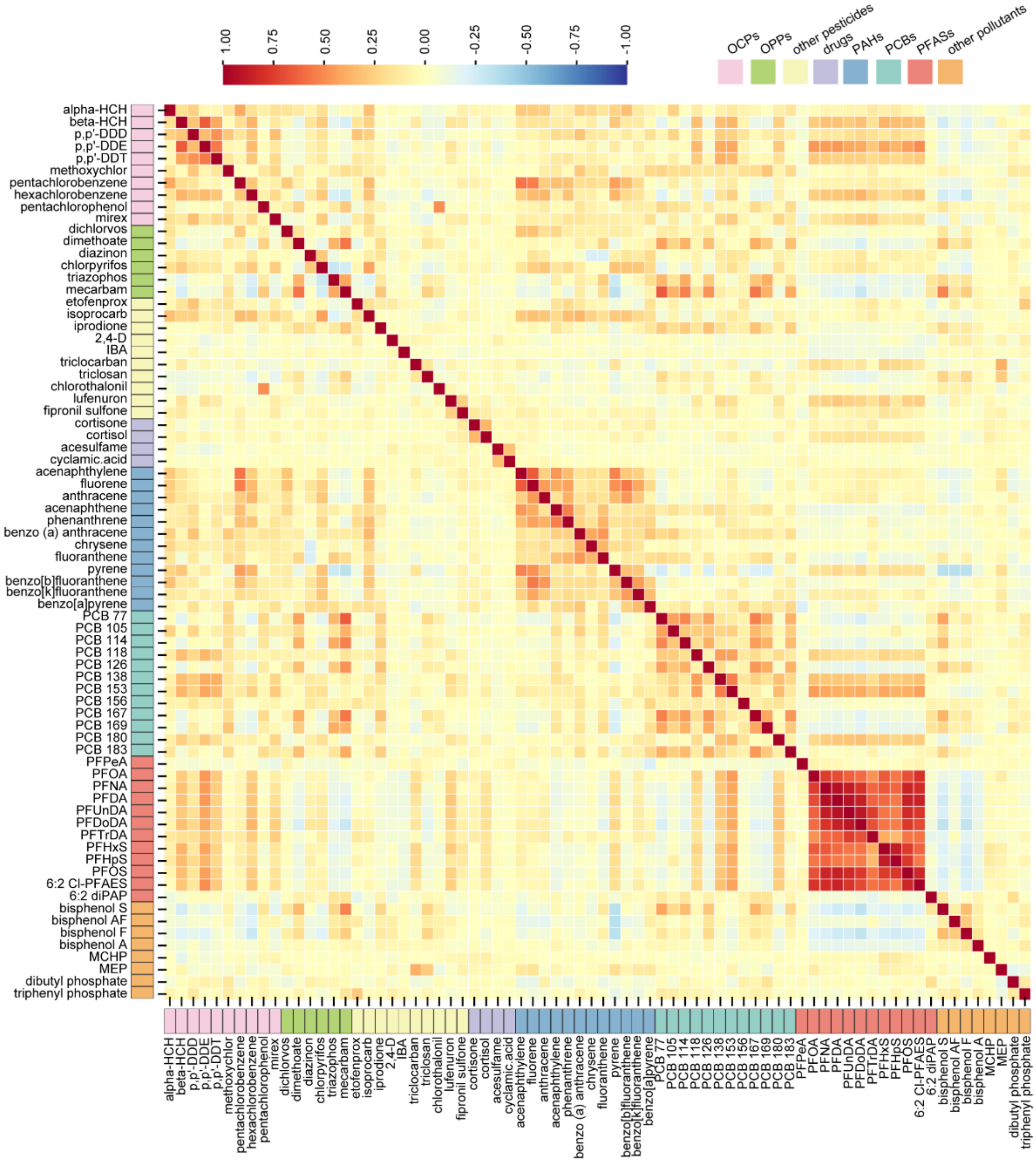

Supplementary Figure 8. Correlations among high-frequency exposures. Pairwise spearman correlations between 74 high-frequency exposures were presented in the heatmap matrix in which red represents positive correlation and blue represents negative correlation. OCP, organochlorine pesticide; OPP, organophosphorus pesticide; PAH, polycyclic aromatic hydrocarbon; PCB, polychlorinated biphenyl; PFAS, perfluoroalkyl substance; HCH, hexachlorocyclohexane; DDD, dichlorodiphenyldichloroethane; DDE, dichlorodiphenyldichloroethylene; DDT, dichlorodiphenyltrichloroethane; IBA, indole-3-butyric acid; PFOA, perfluorooctanoic acid; PFNA, perfluorononanoic acid; PFDA, perfluorodecanoic acid; PFUnDA, perfluoroundecanoic acid; PFDoDA, perfluorododecanoic acid; PFTrDA, perfluorotridecanoic acid; PFOS, perfluorooctanesulfonate; PFHxS, perfluorohexanesulfonate; 6:2 Cl-PFAES, 6:2 chlorinated polyfluoroalkyl ether sulfonate; 6:2 diPAP, bis[2-(perfluorohexyl)ethyl] phosphate; PFPeA, perfluoro-n-pentanoic acid; PFHpS, perfluoroheptanesulfonic acid; MCHP, monocyclohexyl phthalate; MEP, monoethyl phthalate.

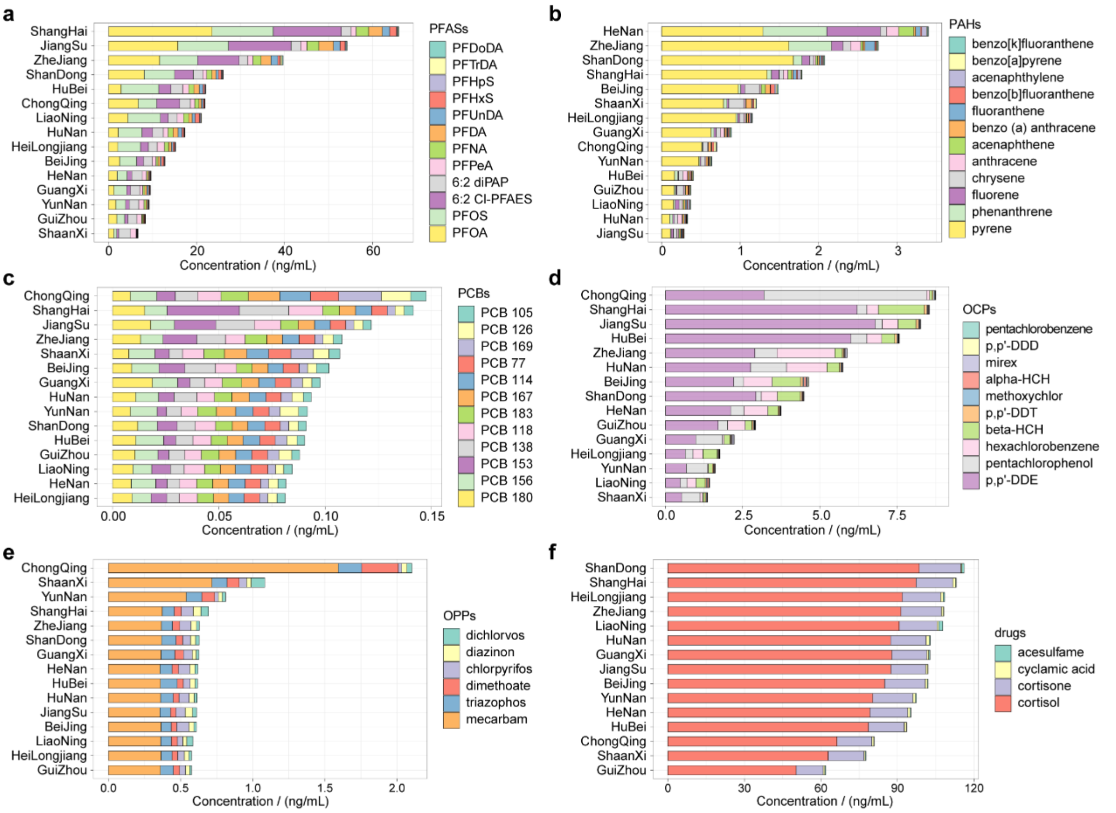

Supplementary Figure 9. Regional distribution of different categories of exposures. Regional distribution of perfluoroalkyl substances (PFASs) (**a**), polycyclic aromatic hydrocarbons (PAHs) (**b**), polychlorinated biphenyl (PCBs) (**c**), organochlorine pesticide (OCPs) (**d**), organophosphorus pesticide (OPPs) (**e**), drugs (**f**). Geometric means of the exposures were used in the bar plot. HCH, hexachlorocyclohexane; DDD, dichlorodiphenyldichloroethane; DDE, dichlorodiphenyldichloroethylene; DDT, dichlorodiphenyltrichloroethane; PFOA, perfluorooctanoic acid; PFNA, perfluorononanoic acid; PFDA, perfluorodecanoic acid; PFUnDA, perfluoroundecanoic acid; PFDoDA, perfluorododecanoic acid; PFTrDA, perfluorotridecanoic acid; PFOS, perfluorooctanesulfonate; PFHxS, perfluorohexanesulfonate; 6:2 Cl-PFAES, 6:2 chlorinated polyfluoroalkyl ether sulfonate; 6:2 diPAP, bis[2-(perfluorohexyl)ethyl] phosphate; PFPeA, perfluoro-n-pentanoic acid; PFHpS, perfluoroheptanesulfonic acid.

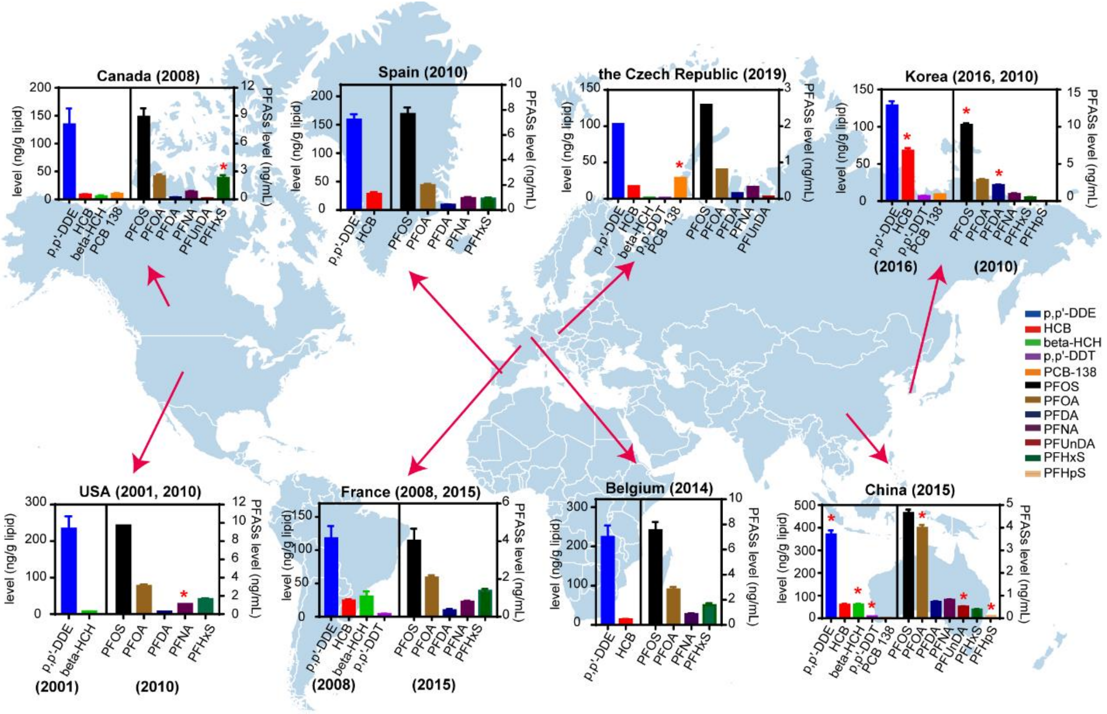

Supplementary Figure 10. The plasma/serum exposures profile in different countries. The comparison of exposure levels includes 8 countries including Belgium, France, USA, Canada, Spain, the Czech Republic, Korea and China. Organochlorine pesticides, polychlorinated biphenyl (PCB)-138 and perfluoroalkyl substances are contained due to widely global data. The columns represent geometric means of concentration, error bar represent 95% confidence interval of geometric means, and * represents the highest exposure level among these 8 countries. DDE, dichlorodiphenyldichloroethylene; HCB, hexachlorobenzene; HCH, hexachlorocyclohexane; DDT, dichlorodiphenyltrichloroethane; PFOS, perfluorooctanesulfonate; PFOA, perfluorooctanoic acid; PFNA, perfluorononanoic acid; PFDA, perfluorodecanoic acid; PFUnDA, perfluoroundecanoic acid; PFHxS, perfluorohexanesulfonate; PFHpS, perfluoroheptanesulfonic acid.

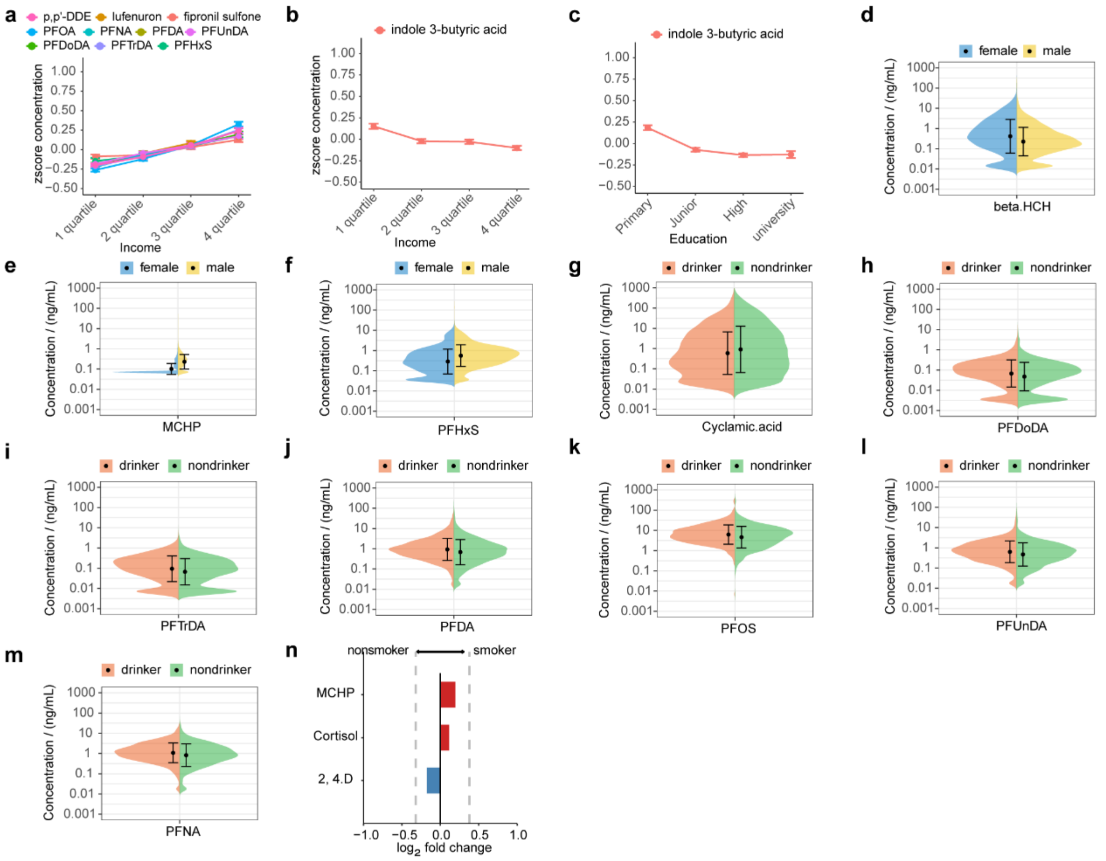

Supplementary Figure 11. Exposure levels in different populations. a Exposures that significantly increase with income levels. Exposures that significantly decrease with income (**b**) and education (**c**) levels. Error bars of figure a-c represent standard error of mean. **d-f** Differential exposures between female and male with fold change of geometric mean less than 0.8 or more than 1.3. **g-m** Differential exposures between drinker and non-drinker with fold change of geometric mean less than 0.8 or more than 1.3. Centre point of violin plot (figure d-m) is the mean, error bars represent standard deviation of mean, outline displays the distribution of the data. **n** Exposures that significantly change with smoking history. Two grey dashed line of figure N represent fold change less than 0.8 and more than 1.3. HCH, hexachlorocyclohexane; DDE, dichlorodiphenyldichloroethylene; PFOA, perfluorooctanoic acid; PFNA, perfluorononanoic acid; PFDA, perfluorodecanoic acid; PFUnDA, perfluoroundecanoic acid; PFDoDA, perfluorododecanoic acid; PFTrDA, perfluorotridecanoic acid; PFOS, perfluorooctanesulfonate; PFHxS, perfluorohexanesulfonate; MCHP, monocyclohexyl phthalate; 2, 4-D, 2,4-Dichlorophenoxyacetic acid.

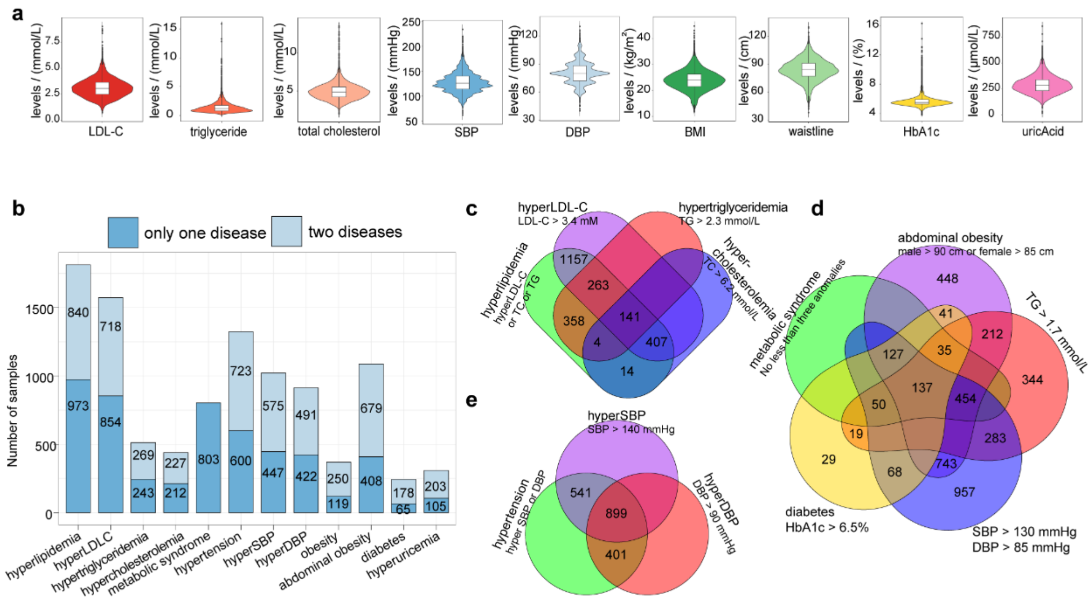

Supplementary Figure 12. Sample information of chronic disease. a Distribution of 9 disease clinical parameters among 5696 individuals. Centre line is the median, box limits indicate upper and lower quartiles, whiskers show 1.5 × interquartile range, points indicate outliers and the outline displays the distribution of the data. **b** Sample size of 12 chronic disease outcomes when considering the combination of one chronic disease. **c-e** Number of samples for hyperlipidemia (c), metabolic syndrome (d), hypertension (e) and their decisive clinical parameters. HbA1c, glycated hemoglobin; LDL-C, low density lipoprotein cholesterol; TC, total cholesterol; TG, triglyceride; SBP, systolic blood pressure; DBP, diastolic blood pressure.

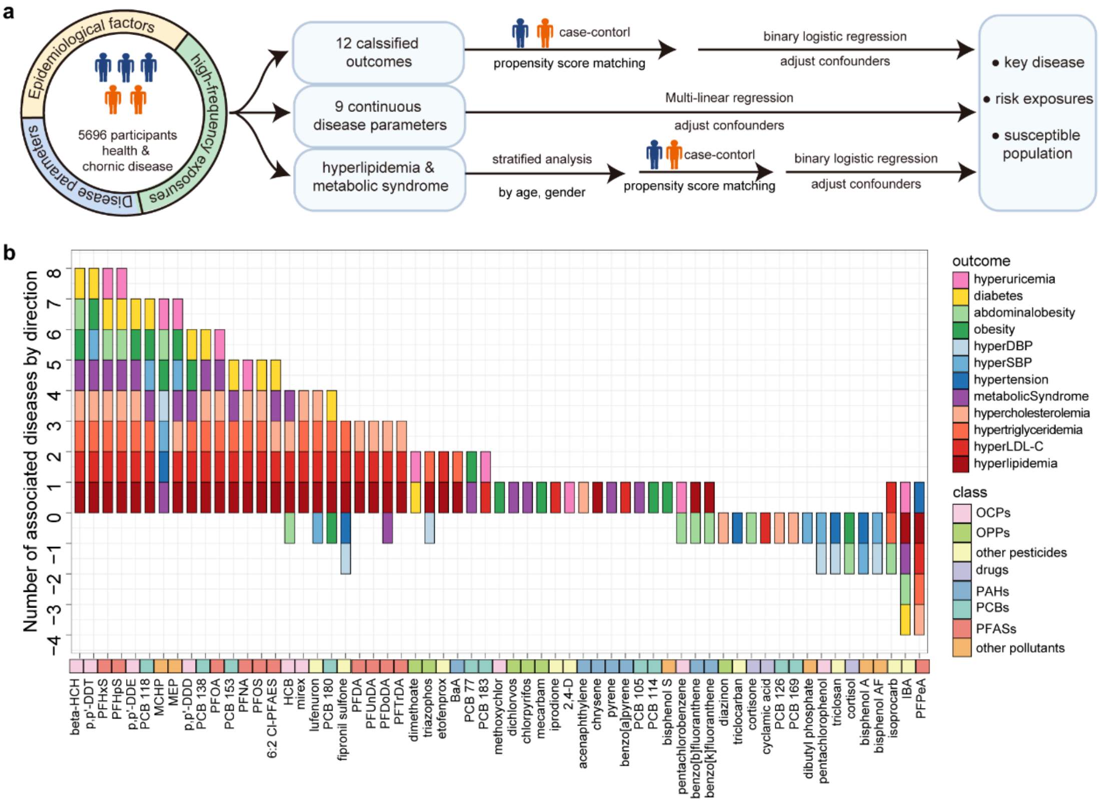

Supplementary Figure 13. The risk of exposure to chronic diseases. a Introduction of 3 analysis methods of the risk for each disease outcomes. **b** Relationship between each exposure and 12 classified disease outcomes. All of regression models adjusted for age, gender, region, sampling time, education and income levels, marital status, smoking and drinking history. Included exposures were significantly associated with at least one disease outcome. LDL-C, low density lipoprotein cholesterol; SBP, systolic blood pressure; DBP, diastolic blood pressure; OCP, organochlorine pesticide; OPP, organophosphorus pesticide; PAH, polycyclic aromatic hydrocarbon; PCB, polychlorinated biphenyl; PFAS, perfluoroalkyl substance. HCH, hexachlorocyclohexane; DDD, dichlorodiphenyldichloroethane; DDE, dichlorodiphenyldichloroethylene; DDT, dichlorodiphenyltrichloroethane; IBA, indole-3-butyric acid; PFOA, perfluorooctanoic acid; PFNA, perfluorononanoic acid; PFDA, perfluorodecanoic acid; PFUnDA, perfluoroundecanoic acid; PFDoDA, perfluorododecanoic acid; PFTrDA, perfluorotridecanoic acid; PFOS, perfluorooctanesulfonate; PFHxS, perfluorohexanesulfonate; 6:2 Cl-PFAES, 6:2 chlorinated polyfluoroalkyl ether sulfonate; 6:2 diPAP, bis[2-(perfluorohexyl)ethyl] phosphate; PFPeA, perfluoro-n-pentanoic acid; PFHpS, perfluoroheptanesulfonic acid; MCHP, monocyclohexyl phthalate; MEP, monoethyl phthalate.

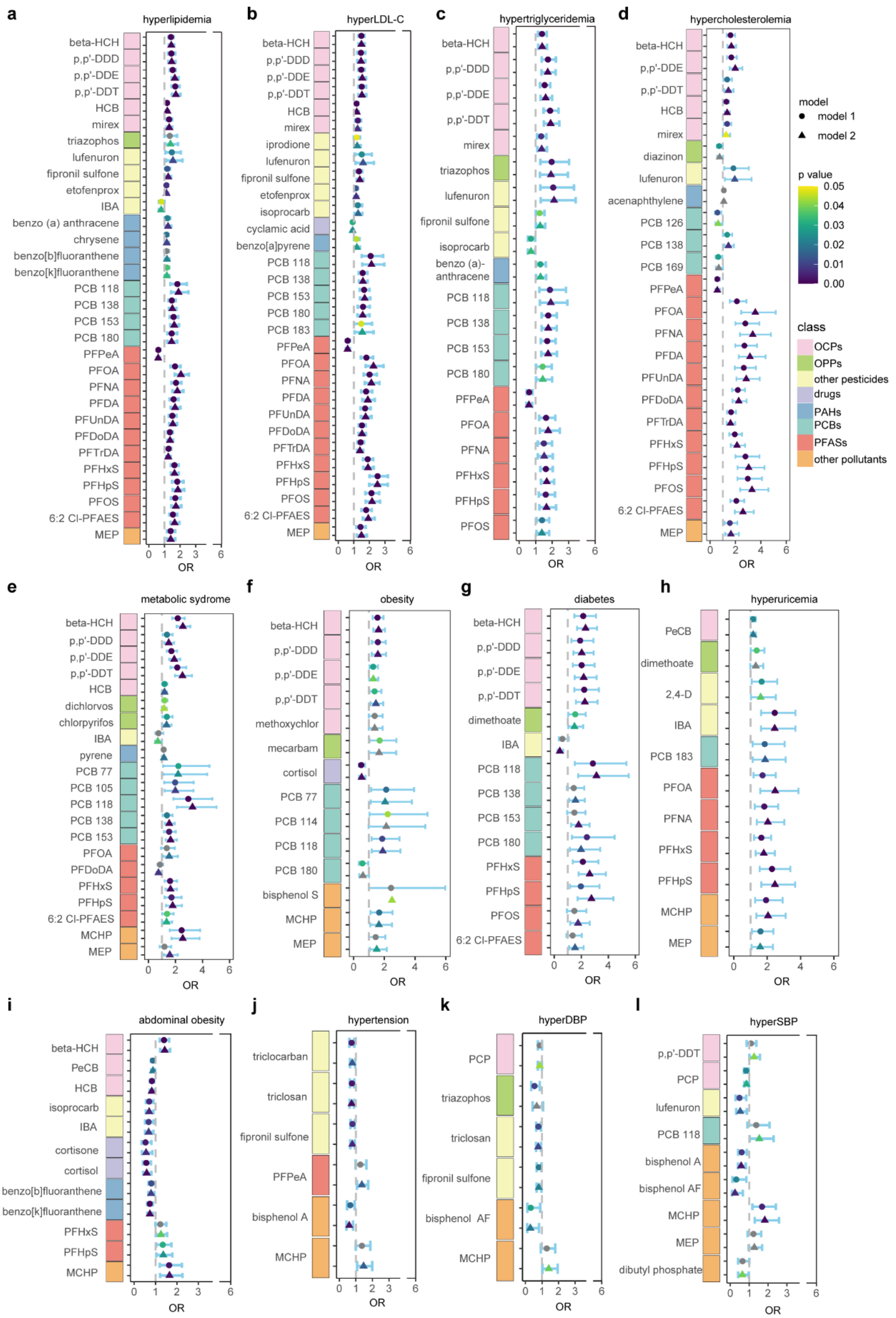

Supplementary Figure 14. The odds ratios of exposure to chronic diseases after considering medical history and external environment factors. Exposures with a significant risk for hyperlipidemia (**a**), hyper low density lipoprotein cholesterol (**b**),

hypercholesterolemia (**c**), hypertriglyceridemia (**d**), metabolic syndrome (**e**), obesity (**f**), diabetes (**g**), hyperuricemia (**h**), abdominal obesity (**i**), hypertension (**j**), hyper diastolic blood pressure (**k**) and hyper systolic blood pressure (**l**). ORs represent odds ratios per one-unit increase in log-transformed exposure levels. Model 1 represents that binary logistic regression models adjusted for age, gender, region, sampling time, education and income levels, marital status, smoking, drinking and medical history (histories of hypertension, diabetes, myocardial infarction, stroke, and cancer). Model 2 represents that binary logistic regression models adjusted for age, gender, region, sampling time, education and income levels, marital status, smoking, drinking history, air pollutions (Air Quality Index (AQI), PM2.5, and PM10) and meteorological conditions (monthly mean values of daily maximum, mean and minimum temperature). The position and colour of circle and triangle represent odds ratios and significant (n = 5696 biologically independent samples, two-sided), respectively. Grey circle or triangle represent no significance. Error bars represent 95% confidence interval of odds ratios.

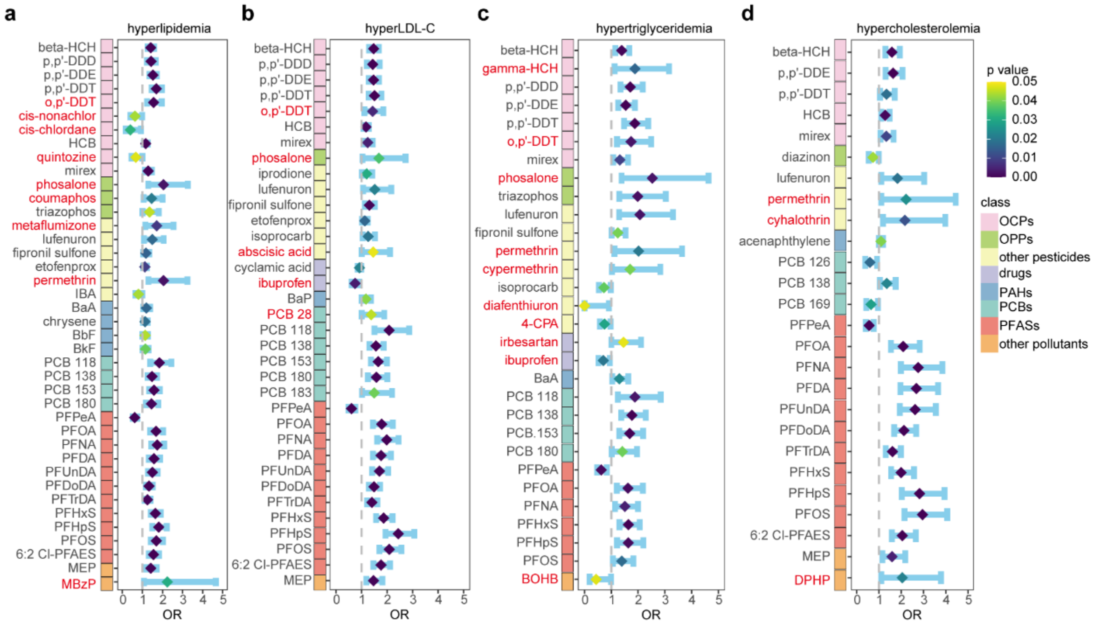

Supplementary Figure 15. The odds ratios of all exposures to chronic diseases (part 1). Exposures with a significant risk for hyperlipidemia (**a**), hyper low density lipoprotein cholesterol (**b**), hypercholesterolemia (**c**), hypertriglyceridemia (**d**). Names in red represent low-frequency exposures. OR represent odds ratio per one-unit increase in log-transformed exposure levels. Binary logistic regression models adjusted for age, gender, region, sampling time, education and income levels, marital status, smoking and drinking history. The position and colour of diamond represent odds ratios and significant (n = 2842 biologically independent samples, two-sided), respectively. Error bars represent 95% confidence interval of odds ratios. PCB, polychlorinated biphenyl; HCH, hexachlorocyclohexane; DDD, dichlorodiphenyldichloroethane; DDE, dichlorodiphenyldichloroethylene; DDT, dichlorodiphenyltrichloroethane; BaA, benzo (a) anthracene; 4-CPA, 4-chlorophenoxyacetate; IBA, indole-3-butyric acid; PFOA, perfluorooctanoic acid; PFNA, perfluorononanoic acid; PFDA, perfluorodecanoic acid; PFUnDA, perfluoroundecanoic acid; PFDoDA, perfluorododecanoic acid; PFTrDA, perfluorotridecanoic acid; PFOS, perfluorooctanesulfonate; PFHxS, perfluorohexanesulfonate; 6:2 Cl-PFAES, 6:2 chlorinated polyfluoroalkyl ether

sulfonate; 6:2 diPAP, bis[2-(perfluorohexyl)ethyl] phosphate; PFPeA, perfluoro-n-pentanoic acid; PFHpS, perfluoroheptanesulfonic acid; MBzP, monobenzyl.phthalate; MEP, monoethyl phthalate; BOHB, n-butyl 4-hydroxybenzoate; DPHP, diphenyl.phosphate.

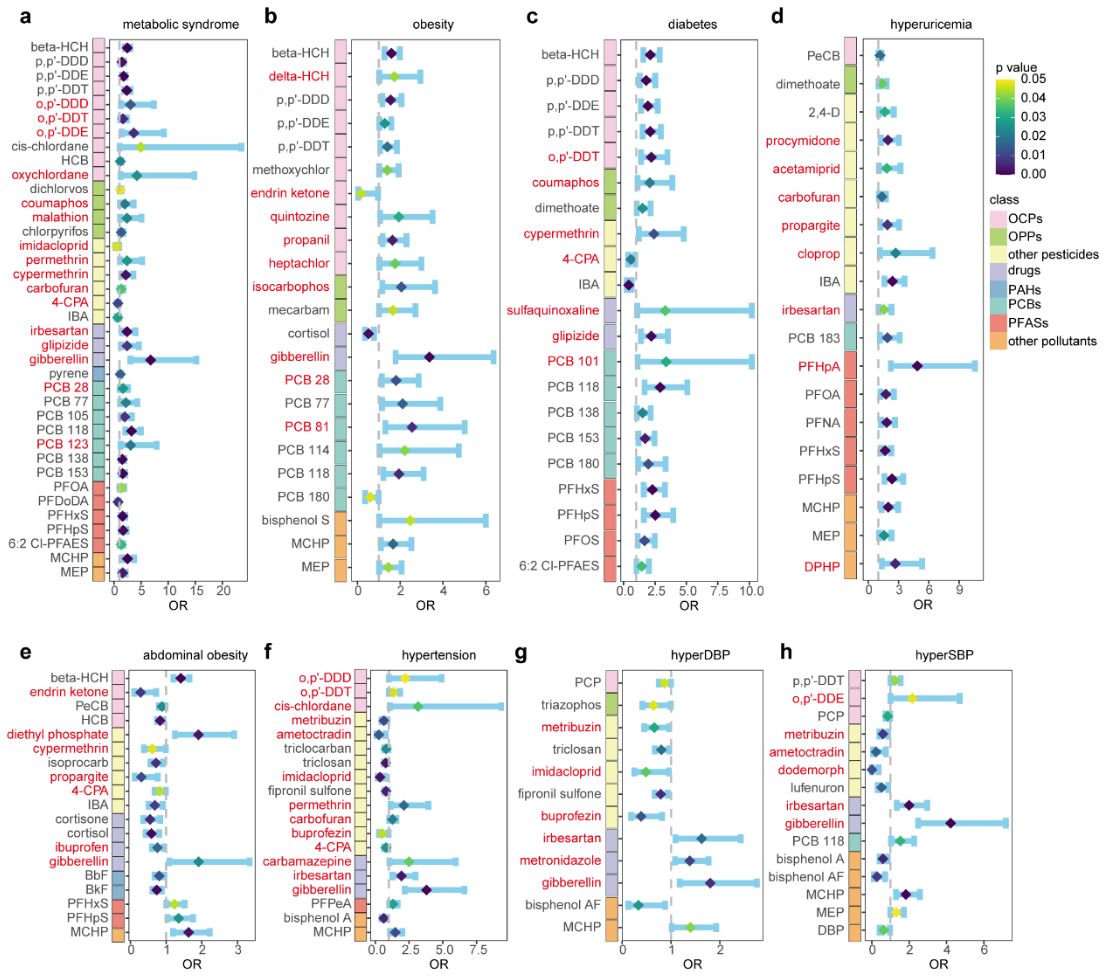

Supplementary Figure 16. The odds ratios of all exposures to chronic diseases (part 2). Exposures with a significant risk for metabolic syndrome (**a**), obesity (**b**), diabetes (**c**), hyperuricemia (**d**), abdominal obesity (**e**), hypertension (**f**), hyper diastolic blood pressure (**g**) and hyper systolic blood pressure (**h**). Names in red represent non-high-frequency exposures. OR represent odds ratio per one-unit increase in log-transformed exposure levels. Binary logistic regression models adjusted for age, gender, region, sampling time, education and income levels, marital status, smoking and drinking history. The position and colour of diamond represent odds ratios and significant (n = 5696 biologically independent samples, two-sided), respectively. Error bars represent 95% confidence interval of odds ratios. PCB, polychlorinated biphenyl; HCH, hexachlorocyclohexane; DDD, dichlorodiphenyldichloroethane; DDE,

dichlorodiphenyldichloroethylene; DDT, dichlorodiphenyltrichloroethane; IBA, indole-3-butyric acid; 4-CPA, 4-chlorophenoxyacetate; PFOA, perfluorooctanoic acid; PFNA, perfluorononanoic acid; PFDA, perfluorodecanoic acid; PFUnDA, perfluoroundecanoic acid; PFDoDA, perfluorododecanoic acid; PFTrDA, perfluorotridecanoic acid; PFOS, perfluorooctanesulfonate; PFHxS, perfluorohexanesulfonate; 6:2 Cl-PFAES, 6:2 chlorinated polyfluoroalkyl ether sulfonate; 6:2 diPAP, bis[2-(perfluorohexyl)ethyl] phosphate; PFPeA, perfluoro-n-pentanoic acid; PFHpS, perfluoroheptanesulfonic acid; MCHP, monocyclohexyl phthalate; MEP, monoethyl phthalate.

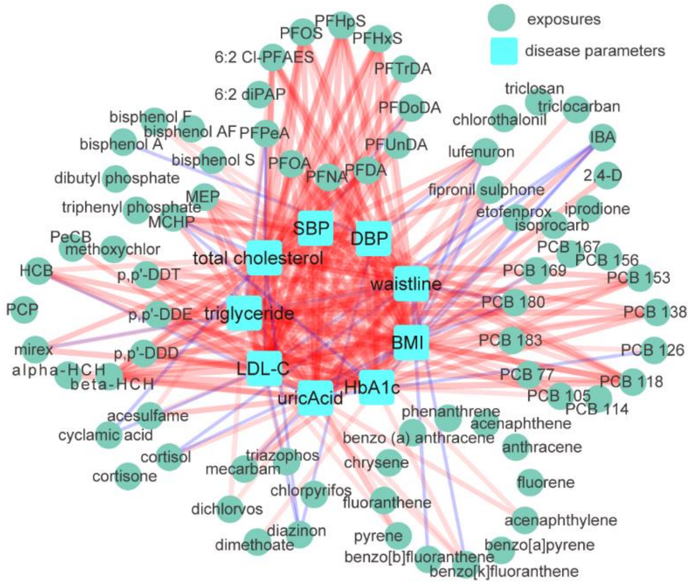

Supplementary Figure 17. Correlation network of high-frequency exposures and 9 clinical disease parameters. Red line represents positive correlation, blue line represents negative correlation obtained by spearman correlation analysis. HbA1c, glycated hemoglobin; BMI, body mass index; LDL-C, low density lipoprotein cholesterol; SBP, systolic blood pressure; DBP, diastolic blood pressure; PCB, polychlorinated biphenyl; HCH, hexachlorocyclohexane; DDD, dichlorodiphenyldichloroethane; DDE, dichlorodiphenyldichloroethylene; DDT, dichlorodiphenyltrichloroethane; IBA, indole-3-butyric acid; PFOA, perfluorooctanoic acid; PFNA, perfluorononanoic acid; PFDA, perfluorodecanoic acid; PFUnDA, perfluoroundecanoic acid; PFDoDA, perfluorododecanoic acid; PFTrDA, perfluorotridecanoic acid; PFOS, perfluorooctanesulfonate; PFHxS, perfluorohexanesulfonate; 6:2 Cl-PFAES, 6:2 chlorinated polyfluoroalkyl ether sulfonate; 6:2 diPAP, bis[2-(perfluorohexyl)ethyl] phosphate; PFPeA, perfluoro-n-pentanoic acid; PFHpS, perfluoroheptanesulfonic acid; MCHP, monocyclohexyl phthalate; MEP, monoethyl phthalate.

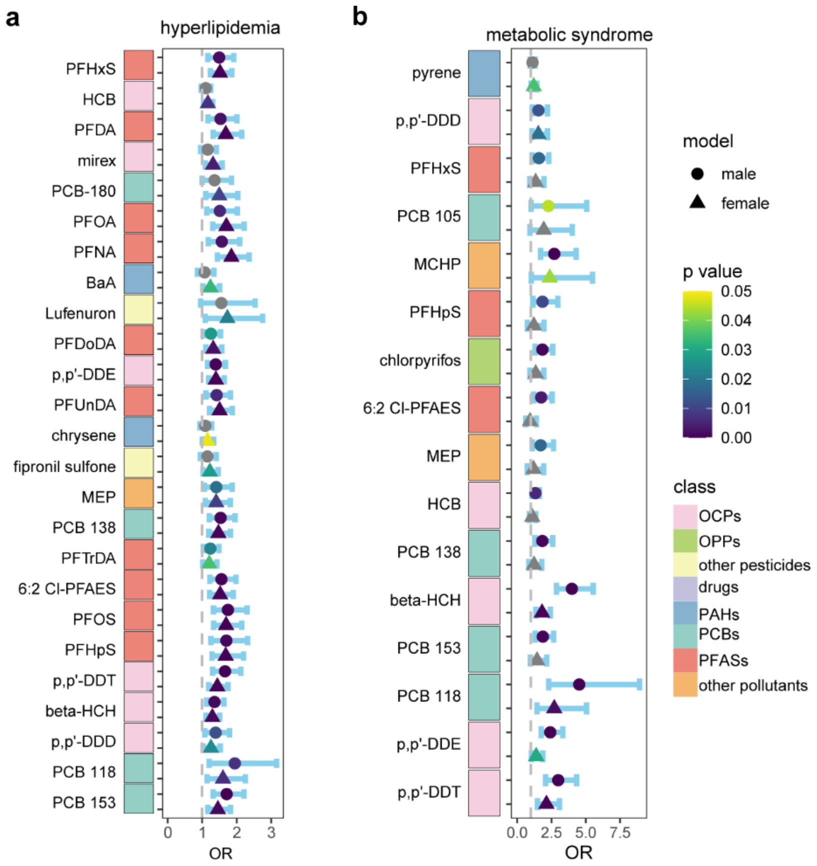

Supplementary Figure 18. Risk analysis stratified by gender. The odds ratios of exposure to hyperlipidemia (**a**) and metabolic syndrome (**b**) stratified by gender. OR represents odds ratio per one-unit increase in log-transformed exposure levels. Binary logistic regression models adjusted for age, gender, region, sampling time, education and income levels, marital status, smoking and drinking history. The position and colour of circle and triangle represent odds ratios and significance (n =2842 biologically independent samples for hyperlipidemia and n =1284 for metabolic syndrome, two-sided), respectively. Grey circle or triangle represents no significance. Error bars represent 95% confidence interval of odds ratios.

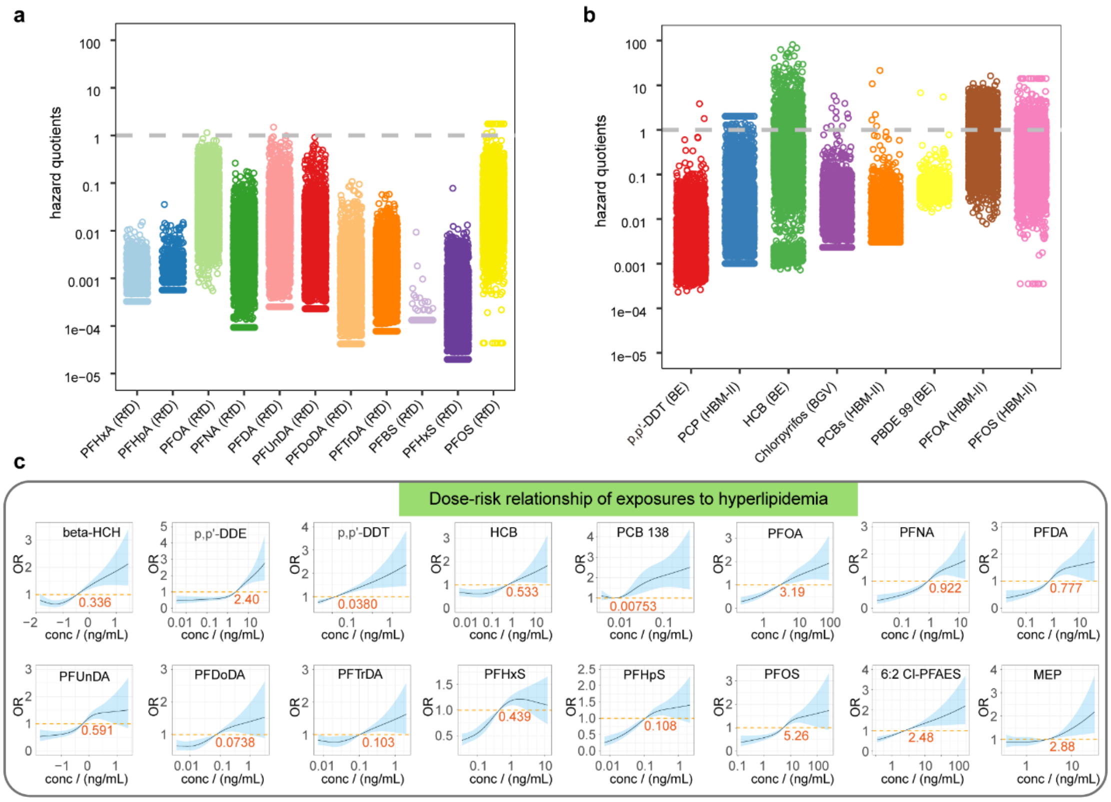

Supplementary Figure 19. Health risk assessment and the determination of exposure guidance values. a Hazard quotients (HQs) for analytes with 11 available reference dose (RfD) values. **b** HQs for analytes with 8 available exposure guidance values including biomonitoring equivalents (BEs), Human Biomonitoring II (HBM II) and Biomonitoring Guidance Values (BGVs). The HQs are the ratios of the observation chemicals concentration of all individuals to the reference value. Each point of jitter_plot represents one individual. **c** Dose-risk relationship of exposures to hyperlipidemia based on data obtained in this study. Chemicals that were significantly associated with the increased risk of hyperlipidemia and showed monotonically increasing does-risk relationship are included. The grey dashed line of figure A, B and orange dashed line of figure C represent health risk threshold. The black solid line represents the odds ratio, and blue shadow represents their 95 % confidence interval.

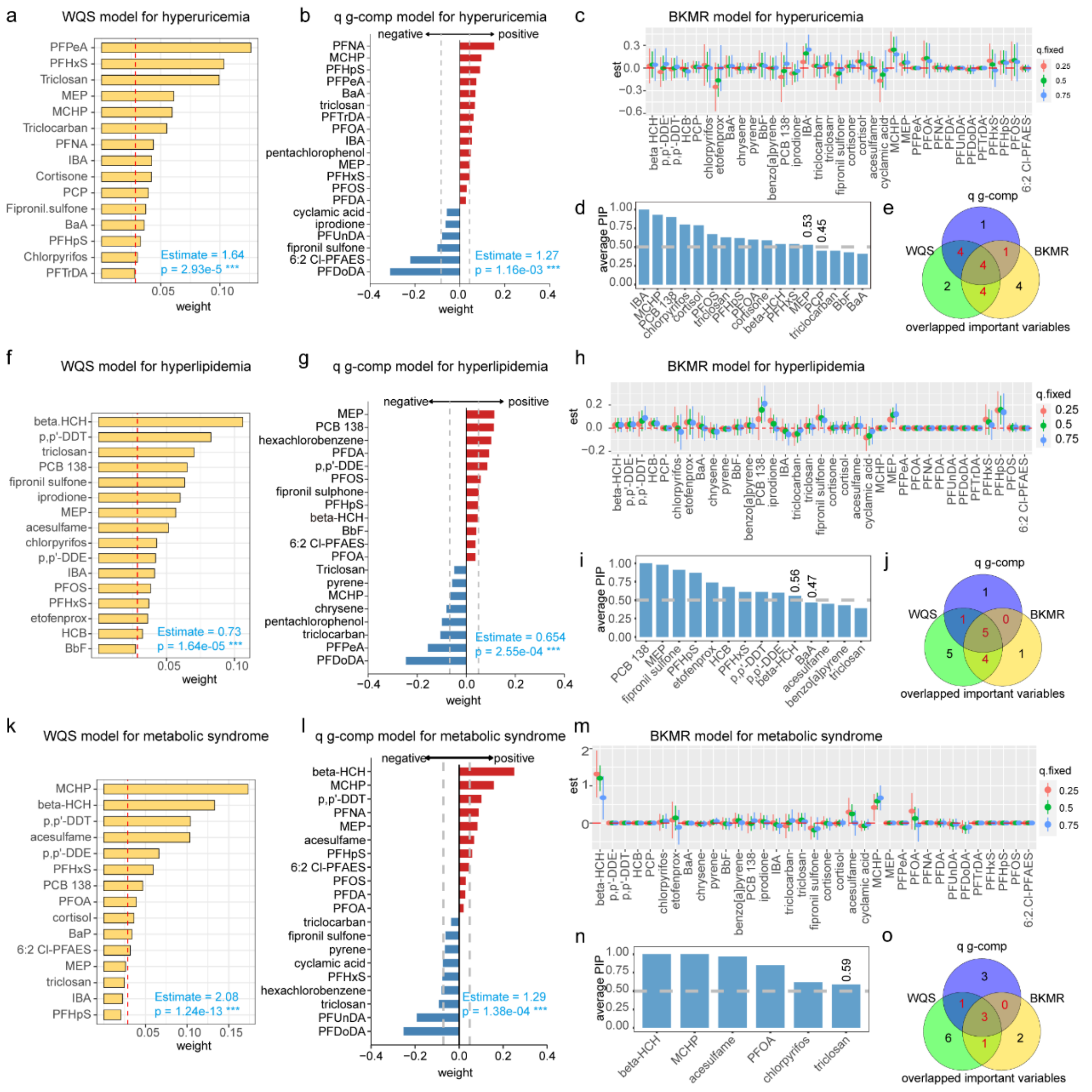

Supplementary Figure 20. The risk of exposure mixtures to chronic diseases. Mixture of exposures that has a positive risk effect on hyperuricemia, based on the model weighted quantile sum regression (WQS) (**a**) and quantile g-computation (q g-comp) (**b**), the dashed line represents mean weight. In Bayesian Kernel Machine Regression (BKMR) Model, individual exposure effect (**c**) and average posterior inclusion probability (PIP) of each exposure which has positive effect (**d**) were determined. **e** Overlapped exposures with significant positive risk effects for hyperuricemia identified by these three models. Numbers with red colour in venn plot represent overlapped exposures. **f-j** Mixture of exposures that has a positive risk effect on hyperlipidemia. **k-o** Mixture of exposures that has a positive risk effect on metabolic syndrome. All of the 3 models adjusted for age, gender, region, smoking and drinking history. HCH, hexachlorocyclohexane; DDD, dichlorodiphenyldichloroethane; DDE,

dichlorodiphenyldichloroethylene; DDT, dichlorodiphenyltrichloroethane; IBA, indole-3-butyric acid; PFOA, perfluorooctanoic acid; PFNA, perfluorononanoic acid; PFDA, perfluorodecanoic acid; PFUnDA, perfluoroundecanoic acid; PFDoDA, perfluorododecanoic acid; PFTrDA, perfluorotridecanoic acid; PFOS, perfluorooctanesulfonate; PFHxS, perfluorohexanesulfonate; 6:2 Cl-PFAES, 6:2 chlorinated polyfluoroalkyl ether sulfonate; 6:2 diPAP, bis[2-(perfluorohexyl)ethyl] phosphate; PFPeA, perfluoro-n-pentanoic acid; PFHpS, perfluoroheptanesulfonic acid; MCHP, monocyclohexyl phthalate; MEP, monoethyl phthalate.

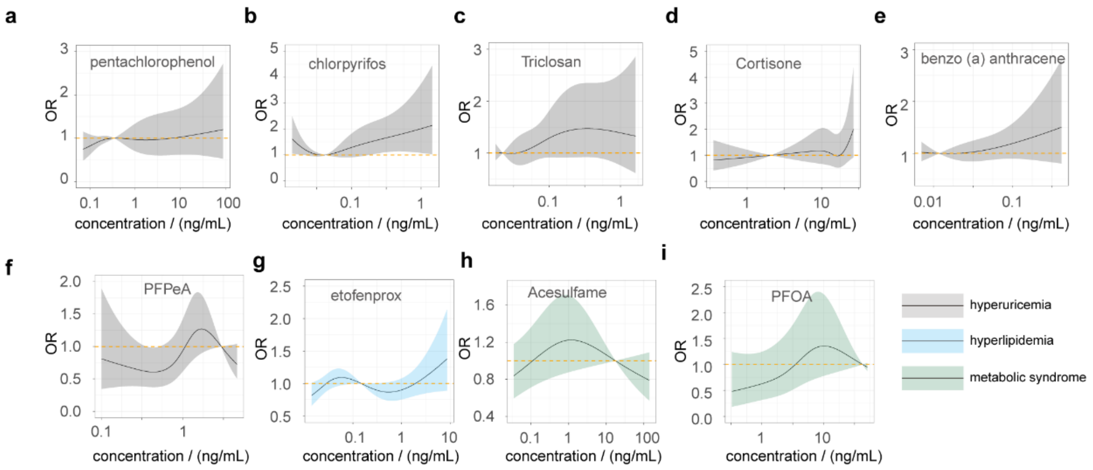

Supplementary Figure 21. Dose-risk relationship between key exposures and related chronic disease. a-f Dose-risk relationship of exposures to hyperuricemia, specifically, 6 key exposures screened by 3 mixture effect models but not significant in binary logistic regression model for analysis of single exposure risk. **g** Dose-risk relationship of exposures to hyperlipidemia, 1 key exposure defined by 3 mixture effect models but not significant in binary logistic regression model for analysis of single exposure risk. **h-i** Dose-risk relationship of exposures to metabolic syndrome, 2 key exposures were only screened by mixture effect models. The black solid line represents the odds ratio, and gray, blue and dark green shadow represents their 95 % confidence interval of hyperuricemia (n = 927 biologically independent samples), hyperlipidemia (n = 2842) and metabolic syndrome (n = 1284), respectively. PFPeA, perfluoro-n-pentanoic acid; PFOA, perfluorooctanoic acid.
