# Supplementary material for: An exposome atlas of serum reveals the risk of chronic diseases in the Chinese population
Source: Nat Commun. 2024 Mar 13;15:2268. doi: 10.1038/s41467-024-46595-z (PMC10937660; doi:10.1038/s41467-024-46595-z)
Supplement: Supplementary file 3 — Description of Additional Supplementary Files [file 41467_2024_46595_MOESM3_ESM.pdf]

Description of Additional Supplementary Files

Supplementary Data 1: Compound information of the total detected 267 exposures.

Supplementary Data 2: The detection frequencies and concentration levels of the 267 exposures in human serum.

Supplementary Data 3: Examination of 29 calibration curve run in GC-MS/MS including recovery for each of the 97 exposures in 2, 5 and 20 ng/mL concentrations.

Supplementary Data 4: Examination of 20 calibration curve run in LC-MS/MS including recovery for each of the 170 exposures in 1, 10 and 100 ng/mL concentrations.

Supplementary Data 5: Targeted method validation based on GC-MS/MS including limit of quantification (LOQ), linear ranges and correlation coefficients (r^2) of calibration curve, recovery, precision for each of the 97 exposures.

Supplementary Data 6: Targeted method validation based on LC-MS/MS including limit of quantification (LOQ), linear ranges and correlation coefficients (r2) of calibration curve, recovery, precision for each of the 170 exposures.

Supplementary Data 7: Sample randomization of the control and each chronic disease group.

Supplementary Data 8: Comparison of concentration levels of common exposures in human blood from 8 countries.

Supplementary Data 9: Sample information in control and each chronic disease after propensity score matching in 4756 samples detected with GC-MS/MS platform.

Supplementary Data 10: Sample information in control and each chronic disease after propensity score matching in 5513 samples detected with LC-MS/MS platform.

Supplementary Data 11: Exposure guidance values for health risk assessment of human biomonitoring data.

Supplementary Data 12: Summary results of the significant relationship between the exposure mixtures and the disease outcomes based on three mixture effect models.

Supplementary Data 13: The review of related literatures on the association between exposures and chronic diseases.

Supplementary Data 14: Information of 8 and 19 internal standards used for GC-MS/MS and LC-MS/MS platform, respectively.

Supplementary Data 15: Optimized GC-MS/MS parameters of 97 exposures including internal standards, retention times (t_R), quantification and qualification transitions and collision energy (CE).

Supplementary Data 16: Optimized LC-MS/MS parameters of 170 exposures including internal standards, transitions, retention times (t_R), collision energy (CE) and declustering potential (DP) voltages.

Supplementary Software: The raw method files of GC-MS/MS and LC-MS/MS.
