# Supplementary material for: An exposome atlas of serum reveals the risk of chronic diseases in the Chinese population
Source: Nat Commun. 2024 Mar 13;15:2268. doi: 10.1038/s41467-024-46595-z (PMC10937660; doi:10.1038/s41467-024-46595-z)
Supplement: Supplementary file 5 — Supplementary Software [file 41467_2024_46595_MOESM5_ESM.zip › method file/GC-MSMS_method.m/acqmethtq.pdf]

Triple Quadrupole Acquisition Method - MS Parameters Report

**Method File**	D:\MassHunter\GCMS\1\methods\Mary1007.m\qqqacqmethod.xml
**Tune File**	atunes.eihs.tune.xml
**Ion Source**	EI
**Source Temp. (° C)**	300
**Electron Energy Mode**	Use tune setting
**Fixed Electron Energy (eV)**	
**Stop Mode**	By Chromatograph Time
**Stop Time (min)**	1
**Solvent Delay (min)**	5
**Use Gain**	Yes
**EM Saver**	1E+08
**Auto Baseline Subtract**	Yes
**Advanced Filtering**	No

Time Filter Table:

**Time filter type: off**		
**Row**	**Time (min)**	**Peak Width (sec)**

Time Segments:

Index	Start Time (min)	Scan Type	Electron Energy (eV)	Data Stored	Delta EMV (V)	Gain
1	5.00	dMRM		X		20

Time Events:

DMRM Statistics:

**Total MRMs**	319
**Minimum Concurrent MRMs**	2
**Maximum Concurrent MRMs**	30
**Min Dwell (group average)**	8.8
**Max Dwell (group average)**	84.9
**Estimated Cycle Time**	256.8353

Compound Table:

Compound Name	ISTD ?	Precursor ion	MS1 res	Product ion	MS2 res	RT	Left Delta RT	Right Delta RT	Dwell (ms)	CE (V)
dichlorvos		185	Wide	93	Wide	6.60	0.35	0.35	63.3	14
dichlorvos		109	Wide	93	Wide	6.60	0.35	0.35	63.3	10
dichlorvos		109	Wide	79	Wide	6.60	0.35	0.35	63.3	8
carbofuran		164.1	Wide	149.1	Wide	7.16	0.48	0.48	63.4	8
carbofuran		164.1	Wide	103.1	Wide	7.16	0.48	0.48	63.4	24
carbofuran		149.1	Wide	77	Wide	7.16	0.48	0.48	63.4	24
acenaphthylene		152.1	Wide	150.1	Wide	9.32	0.47	0.47	51.5	28
acenaphthylene		152.1	Wide	126.1	Wide	9.32	0.47	0.47	51.5	28

Triple Quadrupole Acquisition Method - MS Parameters Report

Compound Table:

Compound Name	ISTD ?	Precursor ion	MS1 res	Product ion	MS2 res	RT	Left Delta RT	Right Delta RT	Dwell (ms)	CE (V)
acenaphthylene		152.1	Wide	102.1	Wide	9.32	0.47	0.47	51.5	28
acenaphthene		153.1	Wide	151.1	Wide	9.83	0.40	0.40	32.5	28
acenaphthene		153.1	Wide	127.1	Wide	9.83	0.40	0.40	32.5	28
acenaphthene		151.1	Wide	77	Wide	9.83	0.40	0.40	32.5	20
pentachlorobenzene		251.9	Wide	216.8	Wide	10.25	0.47	0.47	32.4	22
pentachlorobenzene		249.9	Wide	214.9	Wide	10.25	0.47	0.47	32.4	18
pentachlorobenzene		249.9	Wide	178.9	Wide	10.25	0.47	0.47	32.4	28
isoprocarb		136	Wide	121.1	Wide	10.44	0.44	0.44	51.5	9
isoprocarb		121	Wide	103.1	Wide	10.44	0.44	0.44	51.5	15
isoprocarb		121	Wide	77.1	Wide	10.44	0.44	0.44	51.5	21
fluorene		165.1	Wide	163.1	Wide	11.64	0.45	0.45	84.9	28
fluorene		165.1	Wide	139.1	Wide	11.64	0.45	0.45	84.9	28
fluorene		165.1	Wide	115.1	Wide	11.64	0.45	0.45	84.9	28
trifluralin		306.1	Wide	264.1	Wide	13.03	0.38	0.38	84.7	8
trifluralin		264.1	Wide	206.1	Wide	13.03	0.38	0.38	84.7	8
trifluralin		264.1	Wide	160.1	Wide	13.03	0.38	0.38	84.7	18
α-HCH		218.9	Wide	182.9	Wide	13.88	0.44	0.44	51.4	8
α-HCH		180.9	Wide	144.9	Wide	13.88	0.44	0.44	51.4	16
α-HCH		180.9	Wide	109	Wide	13.88	0.44	0.44	51.4	28
hexachlorobenzene		285.8	Wide	213.8	Wide	14.00	0.47	0.47	37.2	34
hexachlorobenzene		283.8	Wide	248.8	Wide	14.00	0.47	0.47	37.2	24
hexachlorobenzene		283.8	Wide	213.8	Wide	14.00	0.47	0.47	37.2	28
dimethoate		143	Wide	111	Wide	14.46	0.43	0.43	37.3	12
dimethoate		125	Wide	79	Wide	14.46	0.43	0.43	37.3	8
dimethoate		125	Wide	47	Wide	14.46	0.43	0.43	37.3	14
β-HCH		218.9	Wide	182.9	Wide	15.02	0.47	0.47	23.9	8
β-HCH		180.9	Wide	144.9	Wide	15.02	0.47	0.47	23.9	16
β-HCH		180.9	Wide	109	Wide	15.02	0.47	0.47	23.9	28
pentachlorophenol		265.9	Wide	166.9	Wide	15.13	0.46	0.46	20.3	26
pentachlorophenol		265.9	Wide	164.9	Wide	15.13	0.46	0.46	20.3	26
pentachlorophenol		263.9	Wide	164.9	Wide	15.13	0.46	0.46	20.3	30
quintozine		294.8	Wide	264.8	Wide	15.14	0.40	0.40	18.4	12
quintozine		294.8	Wide	236.8	Wide	15.14	0.40	0.40	18.4	16
quintozine		264.8	Wide	236.8	Wide	15.14	0.40	0.40	18.4	10
γ-HCH		218.9	Wide	182.9	Wide	15.40	0.75	0.75	23.3	8
γ-HCH		180.9	Wide	144.9	Wide	15.40	0.75	0.75	23.3	16
γ-HCH		180.9	Wide	109	Wide	15.40	0.75	0.75	23.3	28
diazinon		304.1	Wide	179.1	Wide	16.11	0.34	0.34	19.1	10
diazinon		179.1	Wide	137.1	Wide	16.11	0.34	0.34	19.1	18

Triple Quadrupole Acquisition Method - MS Parameters Report

Compound Table:

Compound Name	ISTD ?	Precursor ion	MS1 res	Product ion	MS2 res	RT	Left Delta RT	Right Delta RT	Dwell (ms)	CE (V)
diazinon		179.1	Wide	122.1	Wide	16.11	0.34	0.34	19.1	24
IS-diazinon	X	153	Wide	138.1	Wide	16.11	0.43	0.43	22.3	9
IS-diazinon	X	138	Wide	85.1	Wide	16.11	0.43	0.43	22.3	15
IS-diazinon	X	138	Wide	55.1	Wide	16.11	0.43	0.43	22.3	27
phenanthrene		178.1	Wide	176.1	Wide	16.12	0.67	0.67	24.4	28
phenanthrene		178.1	Wide	152.1	Wide	16.12	0.67	0.67	24.4	20
phenanthrene		178.1	Wide	150.1	Wide	16.12	0.67	0.67	24.4	42
anthracene		178.1	Wide	176.1	Wide	16.41	0.46	0.46	25.8	28
anthracene		178.1	Wide	152.1	Wide	16.41	0.46	0.46	25.8	20
anthracene		178.1	Wide	150.1	Wide	16.41	0.46	0.46	25.8	42
δ-HCH		218.9	Wide	182.9	Wide	16.82	0.30	0.30	43.8	8
δ-HCH		180.9	Wide	144.9	Wide	16.82	0.30	0.30	43.8	16
δ-HCH		180.9	Wide	109	Wide	16.82	0.30	0.30	43.8	28
propanil		217	Wide	161	Wide	18.18	0.69	0.69	47.8	10
propanil		160.9	Wide	99	Wide	18.18	0.69	0.69	47.8	24
propanil		160.9	Wide	90	Wide	18.18	0.69	0.69	47.8	22
PCB-28		257.9	Wide	186	Wide	18.40	0.62	0.62	35.6	26
PCB-28		255.9	Wide	186	Wide	18.40	0.62	0.62	35.6	26
vinclozolin		285	Wide	212	Wide	18.64	0.49	0.49	35.6	12
vinclozolin		212	Wide	172	Wide	18.64	0.49	0.49	35.6	16
vinclozolin		212	Wide	145	Wide	18.64	0.49	0.49	35.6	24
heptachlor		273.8	Wide	238.9	Wide	19.03	0.45	0.45	57.8	16
heptachlor		271.8	Wide	236.9	Wide	19.03	0.45	0.45	57.8	20
aldrin		292.9	Wide	186	Wide	20.23	0.42	0.42	26.6	40
aldrin		262.9	Wide	193	Wide	20.23	0.42	0.42	26.6	28
aldrin		262.9	Wide	191	Wide	20.23	0.42	0.42	26.6	34
PCB-52		291.9	Wide	221.9	Wide	20.23	0.79	0.79	34.8	26
PCB-52		289.9	Wide	254.9	Wide	20.23	0.79	0.79	34.8	12
PCB-52		289.9	Wide	219.9	Wide	20.23	0.79	0.79	34.8	26
malathion		173.1	Wide	127	Wide	20.86	0.30	0.30	18.4	6
malathion		173.1	Wide	99	Wide	20.86	0.30	0.30	18.4	14
malathion		158.1	Wide	125	Wide	20.86	0.30	0.30	18.4	10
chlorpyrifos		313.9	Wide	285.9	Wide	21.00	0.64	0.64	22	8
chlorpyrifos		313.9	Wide	257.9	Wide	21.00	0.64	0.64	22	14
chlorpyrifos		196.9	Wide	168.9	Wide	21.00	0.64	0.64	22	14
IS-chlorpyrifos	X	200	Wide	171.9	Wide	21.01	0.49	0.49	20.3	15
IS-chlorpyrifos	X	198	Wide	169.9	Wide	21.01	0.49	0.49	20.3	15
IS-chlorpyrifos	X	99	Wide	67	Wide	21.01	0.49	0.49	20.3	15
fenthion		278	Wide	169	Wide	21.25	0.57	0.57	25.8	14

Triple Quadrupole Acquisition Method - MS Parameters Report

Compound Table:

Compound Name	ISTD ?	Precursor ion	MS1 res	Product ion	MS2 res	RT	Left Delta RT	Right Delta RT	Dwell (ms)	CE (V)
fenthion		278	Wide	125	Wide	21.25	0.57	0.57	25.8	20
fenthion		278	Wide	109	Wide	21.25	0.57	0.57	25.8	20
isocarbophos		289.1	Wide	136	Wide	21.70	0.38	0.38	43.7	14
isocarbophos		230	Wide	212	Wide	21.70	0.38	0.38	43.7	10
isocarbophos		230	Wide	198	Wide	21.70	0.38	0.38	43.7	10
oxychlordane		185	Wide	149	Wide	23.17	0.53	0.53	36.8	6
oxychlordane		185	Wide	121	Wide	23.17	0.53	0.53	36.8	12
oxychlordane		185	Wide	85	Wide	23.17	0.53	0.53	36.8	26
heptachlor endo-epoxide		354.8	Wide	219	Wide	23.49	0.30	0.30	20.6	32
heptachlor endo-epoxide		352.8	Wide	253	Wide	23.49	0.30	0.30	20.6	26
heptachlor endo-epoxide		352.8	Wide	219	Wide	23.49	0.30	0.30	20.6	32
chlozolinate		330.9	Wide	258.9	Wide	23.56	0.33	0.33	18.8	6
chlozolinate		258.9	Wide	188	Wide	23.56	0.33	0.33	18.8	14
chlozolinate		258.9	Wide	153	Wide	23.56	0.33	0.33	18.8	28
fluoranthene		202.1	Wide	200.1	Wide	23.65	0.61	0.61	25.1	30
fluoranthene		200.1	Wide	198.1	Wide	23.65	0.61	0.61	25.1	30
mecarbam		329	Wide	131.1	Wide	23.90	0.42	0.42	21.1	18
mecarbam		296	Wide	196.1	Wide	23.90	0.42	0.42	21.1	8
mecarbam		296	Wide	168.1	Wide	23.90	0.42	0.42	21.1	16
procymidone		285	Wide	96	Wide	24.13	0.53	0.53	22.2	10
procymidone		283	Wide	96	Wide	24.13	0.53	0.53	22.2	10
procymidone		283	Wide	68	Wide	24.13	0.53	0.53	22.2	24
cis-chlordane		374.8	Wide	265.9	Wide	24.69	0.47	0.47	19.6	26
cis-chlordane		372.8	Wide	265.9	Wide	24.69	0.47	0.47	19.6	22
cis-chlordane		372.8	Wide	263.9	Wide	24.69	0.47	0.47	19.6	28
methidathion		145	Wide	85	Wide	24.74	0.68	0.68	19.5	8
methidathion		145	Wide	58	Wide	24.74	0.68	0.68	19.5	14
o,p′-DDE		248	Wide	176	Wide	25.01	0.65	0.65	17.1	28
o,p′-DDE		246	Wide	176	Wide	25.01	0.65	0.65	17.1	30
D10-pyrene	X	212	Wide	210	Wide	25.08	0.46	0.46	15.2	33
D10-pyrene	X	212	Wide	208	Wide	25.08	0.46	0.46	15.2	42
D10-pyrene	X	106	Wide	92.1	Wide	25.08	0.46	0.46	15.2	15
pyrene		202.1	Wide	200.1	Wide	25.19	0.59	0.59	16.8	30
pyrene		200.1	Wide	198.1	Wide	25.19	0.59	0.59	16.8	30
PCB-101		325.9	Wide	290.9	Wide	25.25	0.53	0.53	17.3	12
PCB-101		325.9	Wide	255.9	Wide	25.25	0.53	0.53	17.3	26
PCB-101		323.9	Wide	253.9	Wide	25.25	0.53	0.53	17.3	26
trans-chlordane		374.8	Wide	265.9	Wide	25.41	0.44	0.44	17.5	26
trans-chlordane		372.8	Wide	265.9	Wide	25.41	0.44	0.44	17.5	22

Triple Quadrupole Acquisition Method - MS Parameters Report

Compound Table:

Compound Name	ISTD ?	Precursor ion	MS1 res	Product ion	MS2 res	RT	Left Delta RT	Right Delta RT	Dwell (ms)	CE (V)
trans-chlordane		372.8	Wide	263.9	Wide	25.41	0.44	0.44	17.5	28
trans-nonachlor		406.8	Wide	334.9	Wide	25.58	0.47	0.47	31.1	16
trans-nonachlor		406.8	Wide	299.9	Wide	25.58	0.47	0.47	31.1	24
trans-nonachlor		406.8	Wide	109	Wide	25.58	0.47	0.47	31.1	22
PCB-81		291.9	Wide	221.9	Wide	26.58	0.62	0.62	34.3	26
PCB-81		289.9	Wide	219.9	Wide	26.58	0.62	0.62	34.3	26
p,p'-DDE-13C12	X	330	Wide	258	Wide	26.69	0.62	0.62	29.7	21
p,p'-DDE-13C12	X	260	Wide	188.1	Wide	26.69	0.65	0.65	30.1	30
p,p'-DDE-13C12	X	258	Wide	188	Wide	26.69	0.65	0.65	30.1	39
p,p′-DDE		317.9	Wide	248	Wide	26.70	0.35	0.35	26.3	24
p,p′-DDE		246	Wide	176	Wide	26.70	0.35	0.35	26.3	30
o,p′-DDD		237	Wide	165	Wide	26.97	0.35	0.35	24.8	28
o,p′-DDD		235	Wide	199	Wide	26.97	0.55	0.55	26.4	16
o,p′-DDD		235	Wide	165	Wide	26.97	0.55	0.55	26.4	24
PCB-77		291.9	Wide	221.9	Wide	27.05	0.62	0.62	30.2	26
PCB-77		289.9	Wide	219.9	Wide	27.05	0.62	0.62	30.2	26
chlorfenapyr		247.1	Wide	227	Wide	27.57	0.30	0.30	34.7	16
chlorfenapyr		247.1	Wide	200	Wide	27.57	0.30	0.30	34.7	24
chlorfenapyr		247.1	Wide	75	Wide	27.57	0.30	0.30	34.7	24
PCB-123		325.9	Wide	255.9	Wide	27.94	0.30	0.30	21.9	26
PCB-123		323.9	Wide	253.9	Wide	27.94	0.30	0.30	21.9	26
PCB-118		325.9	Wide	255.9	Wide	28.10	0.39	0.39	15.7	26
PCB-118		323.9	Wide	253.9	Wide	28.10	0.39	0.39	15.7	26
β-endosulfan		194.9	Wide	160	Wide	28.10	0.37	0.37	14.2	8
β-endosulfan		194.9	Wide	125	Wide	28.10	0.37	0.37	14.2	24
β-endosulfan		194.9	Wide	123	Wide	28.10	0.37	0.37	14.2	22
endrin		262.9	Wide	228	Wide	28.11	0.37	0.37	14.2	22
endrin		262.9	Wide	193	Wide	28.11	0.37	0.37	14.2	28
endrin		262.9	Wide	191	Wide	28.11	0.37	0.37	14.2	30
PBDE-28		407.8	Wide	247.9	Wide	28.30	0.37	0.37	10.5	20
PBDE-28		405.8	Wide	247.9	Wide	28.30	0.37	0.37	10.5	20
PBDE-28		405.8	Wide	245.9	Wide	28.30	0.37	0.37	10.5	20
cis-nonachlor		408.8	Wide	145	Wide	28.31	0.41	0.41	11.3	24
cis-nonachlor		406.8	Wide	299.9	Wide	28.31	0.41	0.41	11.3	24
cis-nonachlor		406.8	Wide	109	Wide	28.31	0.41	0.41	11.3	22
PCB-114		325.9	Wide	255.9	Wide	28.45	0.41	0.41	11.1	26
PCB-114		323.9	Wide	253.9	Wide	28.45	0.41	0.41	11.1	26
p,p'-DDD-13C12	X	249	Wide	177.1	Wide	28.46	0.33	0.33	9.9	27
p,p'-DDD-13C12	X	247	Wide	211.1	Wide	28.46	0.33	0.33	9.9	18

Triple Quadrupole Acquisition Method - MS Parameters Report

Compound Table:

Compound Name	ISTD ?	Precursor ion	MS1 res	Product ion	MS2 res	RT	Left Delta RT	Right Delta RT	Dwell (ms)	CE (V)
p,p'-DDD-13C12	X	247	Wide	177.1	Wide	28.46	0.33	0.33	9.9	33
o,p`-DDT		237	Wide	165	Wide	28.47	0.30	0.30	9.3	28
o,p`-DDT		235	Wide	199	Wide	28.47	0.30	0.30	9.3	16
o,p`-DDT		235	Wide	165	Wide	28.47	0.30	0.30	9.3	24
p,p′-DDD		237	Wide	165	Wide	28.47	0.31	0.31	9.8	28
p,p′-DDD		235	Wide	199	Wide	28.47	0.31	0.31	9.8	16
p,p′-DDD		235	Wide	165	Wide	28.47	0.31	0.31	9.8	24
ethion		230.9	Wide	174.9	Wide	28.58	0.36	0.36	12.3	14
ethion		230.9	Wide	129	Wide	28.58	0.36	0.36	12.3	24
ethion		153	Wide	97	Wide	28.58	0.36	0.36	12.3	14
PCB-153		361.9	Wide	291.9	Wide	28.92	0.44	0.44	16.5	28
PCB-153		359.9	Wide	324.9	Wide	28.92	0.44	0.44	16.5	14
PCB-153		359.9	Wide	289.9	Wide	28.92	0.44	0.44	16.5	28
PCB-105		325.9	Wide	255.9	Wide	29.02	0.48	0.48	19.5	26
PCB-105		323.9	Wide	253.9	Wide	29.02	0.48	0.48	19.5	26
triazophos		257	Wide	162	Wide	29.15	0.41	0.41	21.9	8
triazophos		161	Wide	134	Wide	29.15	0.41	0.41	21.9	8
triazophos		161	Wide	91	Wide	29.15	0.41	0.41	21.9	18
p,p'-DDT-13C12	X	249	Wide	177.1	Wide	29.81	0.30	0.30	22.5	27
p,p'-DDT-13C12	X	247	Wide	211.2	Wide	29.81	0.30	0.30	22.5	18
p,p'-DDT-13C12	X	247	Wide	177.1	Wide	29.81	0.30	0.30	22.5	24
p,p′-DDT		237	Wide	165	Wide	29.82	0.40	0.40	22.3	28
p,p′-DDT		235	Wide	165	Wide	29.82	0.40	0.40	22.3	24
PCB-138		361.9	Wide	291.9	Wide	29.82	0.50	0.50	23.2	28
PCB-138		359.9	Wide	324.9	Wide	29.82	0.50	0.50	23.2	14
PCB-138		359.9	Wide	289.9	Wide	29.82	0.50	0.50	23.2	28
PCB-126		325.9	Wide	255.9	Wide	30.18	0.43	0.43	22.3	26
PCB-126		323.9	Wide	253.9	Wide	30.18	0.43	0.43	22.3	26
propargite		135.1	Wide	107.1	Wide	30.46	0.42	0.42	24.1	16
propargite		135.1	Wide	95	Wide	30.46	0.42	0.42	24.1	14
propargite		135.1	Wide	77	Wide	30.46	0.42	0.42	24.1	24
PCB-183		395.8	Wide	325.9	Wide	30.49	0.47	0.47	22.4	28
PCB-183		393.8	Wide	358.8	Wide	30.49	0.47	0.47	22.4	14
PCB-183		393.8	Wide	323.9	Wide	30.49	0.47	0.47	22.4	28
PCB-167		361.9	Wide	291.9	Wide	30.78	0.39	0.39	21.2	28
PCB-167		359.9	Wide	289.9	Wide	30.78	0.39	0.39	21.2	28
endrin ketone		317	Wide	245	Wide	31.08	0.30	0.30	15.3	15
endrin ketone		317	Wide	101	Wide	31.08	0.30	0.30	15.3	25
iprodione		316	Wide	56	Wide	31.21	0.30	0.30	12.2	24

Triple Quadrupole Acquisition Method - MS Parameters Report

Compound Table:

Compound Name	ISTD ?	Precursor ion	MS1 res	Product ion	MS2 res	RT	Left Delta RT	Right Delta RT	Dwell (ms)	CE (V)
iprodione		314	Wide	245	Wide	31.21	0.30	0.30	12.2	12
iprodione		314	Wide	56	Wide	31.21	0.30	0.30	12.2	22
benz(a)anthracene		228.1	Wide	226.1	Wide	31.33	0.39	0.39	11.2	32
benz(a)anthracene		228.1	Wide	224.1	Wide	31.33	0.39	0.39	11.2	60
benz(a)anthracene		226.1	Wide	224.1	Wide	31.33	0.39	0.39	11.2	32
PCB-156-D3		364.7	Wide	295	Wide	31.43	0.30	0.30	10.1	35
PCB-156-D3		362.8	Wide	292.8	Wide	31.43	0.30	0.30	10.1	30
PCB-156-D3		292.3	Wide	221.1	Wide	31.43	0.30	0.30	10.1	35
PCB-156		361.9	Wide	291.9	Wide	31.43	0.39	0.39	10.5	28
PCB-156		359.9	Wide	289.9	Wide	31.43	0.39	0.39	10.5	28
chrysene		228.1	Wide	226.1	Wide	31.45	0.56	0.56	12.2	32
chrysene		228.1	Wide	224.1	Wide	31.45	0.56	0.56	12.2	60
chrysene		226.1	Wide	224.1	Wide	31.45	0.56	0.56	12.2	32
bifenthrin		181.1	Wide	179.1	Wide	31.52	0.32	0.32	9	12
bifenthrin		181.1	Wide	166.1	Wide	31.52	0.32	0.32	9	12
bifenthrin		181.1	Wide	153.1	Wide	31.52	0.32	0.32	9	8
PCB-157		361.9	Wide	326.9	Wide	31.57	0.38	0.38	9.5	14
PCB-157		361.9	Wide	291.9	Wide	31.57	0.30	0.30	8.8	28
PCB-157		359.9	Wide	324.9	Wide	31.57	0.30	0.30	8.8	14
PCB-157		359.9	Wide	289.9	Wide	31.57	0.30	0.30	8.8	28
tetramethrin		164.1	Wide	107.1	Wide	31.61	0.36	0.36	9.6	14
tetramethrin		164.1	Wide	77	Wide	31.61	0.36	0.36	9.6	22
tetramethrin		123.1	Wide	81	Wide	31.61	0.36	0.36	9.6	8
methoxychlor		227.1	Wide	212.1	Wide	31.65	0.31	0.31	9.6	14
methoxychlor		227.1	Wide	169.1	Wide	31.65	0.31	0.31	9.6	24
methoxychlor		227.1	Wide	141.1	Wide	31.65	0.31	0.31	9.6	28
fenpropathrin		265.1	Wide	210.1	Wide	31.79	0.38	0.38	12.4	12
fenpropathrin		265.1	Wide	89	Wide	31.79	0.38	0.38	12.4	28
fenpropathrin		181.1	Wide	152.1	Wide	31.79	0.38	0.38	12.4	22
PCB-180		395.8	Wide	325.9	Wide	31.91	0.39	0.39	13.9	28
PCB-180		393.8	Wide	358.8	Wide	31.91	0.39	0.39	13.9	14
PCB-180		393.8	Wide	323.9	Wide	31.91	0.39	0.39	13.9	28
PBDE-47		487.7	Wide	327.7	Wide	32.09	0.36	0.36	17.1	24
PBDE-47		487.7	Wide	325.7	Wide	32.09	0.36	0.36	17.1	24
PBDE-47		485.7	Wide	325.7	Wide	32.09	0.36	0.36	17.1	24
phosalone		182	Wide	138	Wide	32.40	0.34	0.34	27.5	9
phosalone		182	Wide	111	Wide	32.40	0.34	0.34	27.5	15
phosalone		182	Wide	75	Wide	32.40	0.34	0.34	27.5	33
PCB-169		361.9	Wide	291.9	Wide	32.52	0.45	0.45	26	28

Triple Quadrupole Acquisition Method - MS Parameters Report

Compound Table:

Compound Name	ISTD ?	Precursor ion	MS1 res	Product ion	MS2 res	RT	Left Delta RT	Right Delta RT	Dwell (ms)	CE (V)
PCB-169		359.9	Wide	289.9	Wide	32.52	0.45	0.45	26	28
cyhalothrin-1		197	Wide	141.1	Wide	32.78	0.30	0.30	22.5	15
cyhalothrin-1		181	Wide	152.1	Wide	32.78	0.30	0.30	22.5	30
cyhalothrin-1		181	Wide	127.1	Wide	32.78	0.30	0.30	22.5	30
mires		273.8	Wide	236.8	Wide	32.93	0.42	0.42	22.5	18
mirex		273.8	Wide	238.8	Wide	32.93	0.42	0.42	22.5	18
mirex		271.8	Wide	236.8	Wide	32.93	0.42	0.42	22.5	18
cyhalothrin-2		197	Wide	141.1	Wide	33.04	0.37	0.37	27.2	15
cyhalothrin-2		181	Wide	152.1	Wide	33.04	0.37	0.37	27.2	30
cyhalothrin-2		181	Wide	127.1	Wide	33.04	0.37	0.37	27.2	30
PCB-189		395.8	Wide	325.9	Wide	33.48	0.53	0.53	47.5	28
PCB-189		393.8	Wide	323.9	Wide	33.48	0.53	0.53	47.5	28
permethrin(trans)		183.1	Wide	168.1	Wide	34.15	0.30	0.30	36	14
permethrin(trans)		183.1	Wide	165.1	Wide	34.15	0.30	0.30	36	14
permethrin(trans)		183.1	Wide	153.1	Wide	34.15	0.30	0.30	36	14
coumaphos		362	Wide	226	Wide	34.28	0.30	0.30	28.3	14
coumaphos		362	Wide	109	Wide	34.28	0.30	0.30	28.3	16
coumaphos		226	Wide	163	Wide	34.28	0.30	0.30	28.3	18
permethrin(cis)		183.1	Wide	168.1	Wide	34.34	0.30	0.30	24.1	14
permethrin(cis)		183.1	Wide	165.1	Wide	34.34	0.30	0.30	24.1	14
permethrin(cis)		183.1	Wide	153.1	Wide	34.34	0.30	0.30	24.1	14
PBDE-99		565.6	Wide	405.7	Wide	34.36	0.32	0.32	27.6	26
PBDE-99		563.6	Wide	405.7	Wide	34.36	0.32	0.32	27.6	26
PBDE-99		563.6	Wide	403.7	Wide	34.36	0.32	0.32	27.6	26
benzo(b)fluoranthene		252.1	Wide	250.1	Wide	34.98	0.48	0.48	21.6	36
benzo(b)fluoranthene		252.1	Wide	248.1	Wide	34.98	0.48	0.48	21.6	60
benzo(b)fluoranthene		250.1	Wide	248.1	Wide	34.98	0.48	0.48	21.6	36
PBDE-100		565.6	Wide	405.7	Wide	35.05	0.30	0.30	15.3	26
PBDE-100		563.6	Wide	405.7	Wide	35.05	0.30	0.30	15.3	26
PBDE-100		563.6	Wide	403.7	Wide	35.05	0.30	0.30	15.3	26
cyfluthrin-1		226.1	Wide	206.1	Wide	35.06	0.30	0.30	14.5	14
cyfluthrin-1		163.1	Wide	127.1	Wide	35.06	0.30	0.30	14.5	6
cyfluthrin-1		163.1	Wide	91	Wide	35.06	0.30	0.30	14.5	14
benzo(k)fluoranthene		252.1	Wide	250.1	Wide	35.07	0.30	0.30	12.6	36
benzo(k)fluoranthene		252.1	Wide	248.1	Wide	35.07	0.30	0.30	12.6	60
benzo(k)fluoranthene		250.1	Wide	248.1	Wide	35.07	0.30	0.30	12.6	36
cyfluthrin-2		226.1	Wide	206.1	Wide	35.14	0.30	0.30	11.1	14
cyfluthrin-2		163.1	Wide	127.1	Wide	35.14	0.30	0.30	11.1	6
cyfluthrin-2		163.1	Wide	91	Wide	35.14	0.30	0.30	11.1	14

Compound Table:

Compound Name	ISTD ?	Precursor ion	MS1 res	Product ion	MS2 res	RT	Left Delta RT	Right Delta RT	Dwell (ms)	CE (V)
cyfluthrin-3		226.1	Wide	206.1	Wide	35.20	0.30	0.30	10.9	14
cyfluthrin-3		163.1	Wide	127.1	Wide	35.20	0.30	0.30	10.9	6
cyfluthrin-3		163.1	Wide	91	Wide	35.20	0.30	0.30	10.9	14
cypermethrin-1		181.1	Wide	152.1	Wide	35.51	0.30	0.30	10.6	22
cypermethrin-1		163.1	Wide	127.1	Wide	35.51	0.30	0.30	10.6	6
cypermethrin-1		163.1	Wide	91	Wide	35.51	0.30	0.30	10.6	14
cypermethrin-2		181.1	Wide	152.1	Wide	35.57	0.30	0.30	11.2	22
cypermethrin-2		163.1	Wide	127.1	Wide	35.57	0.30	0.30	11.2	6
cypermethrin-2		163.1	Wide	91	Wide	35.57	0.30	0.30	11.2	14
cypermethrin-3		181.1	Wide	152.1	Wide	35.63	0.31	0.31	12.4	22
cypermethrin-3		163.1	Wide	127.1	Wide	35.63	0.31	0.31	12.4	6
cypermethrin-3		163.1	Wide	91	Wide	35.63	0.31	0.31	12.4	14
etofenprox		163	Wide	135.1	Wide	35.79	0.39	0.39	19.1	9
etofenprox		163	Wide	107.1	Wide	35.79	0.39	0.39	19.1	21
etofenprox		135	Wide	107.1	Wide	35.79	0.39	0.39	19.1	9
IS-etofenprox	X	168	Wide	136.1	Wide	35.79	0.31	0.31	19	12
IS-etofenprox	X	168	Wide	108.1	Wide	35.79	0.31	0.31	19	21
IS-etofenprox	X	136	Wide	108.1	Wide	35.79	0.31	0.31	19	12
benzo(a)pyrene		252.1	Wide	250.1	Wide	35.87	0.56	0.56	23.3	36
benzo(a)pyrene		252.1	Wide	248.1	Wide	35.87	0.56	0.56	23.3	60
benzo(a)pyrene		250.1	Wide	248.1	Wide	35.87	0.56	0.56	23.3	36
fenvalerate-1		419.1	Wide	225.1	Wide	36.54	0.33	0.33	29.7	6
fenvalerate-1		225.1	Wide	147.1	Wide	36.54	0.33	0.33	29.7	10
fenvalerate-1		225.1	Wide	119.1	Wide	36.54	0.33	0.33	29.7	20
fenvalerate-1		225.1	Wide	91	Wide	36.54	0.33	0.33	29.7	26
PBDE 153		645.6	Wide	485.7	Wide	36.65	0.30	0.30	29.7	26
PBDE 153		645.6	Wide	483.7	Wide	36.65	0.30	0.30	29.7	26
PBDE 153		643.6	Wide	483.7	Wide	36.65	0.30	0.30	29.7	26
fenvalerate-2		419.1	Wide	225.1	Wide	36.82	0.30	0.30	40.5	6
fenvalerate-2		225.1	Wide	147.1	Wide	36.82	0.30	0.30	40.5	10
fenvalerate-2		225.1	Wide	119.1	Wide	36.82	0.30	0.30	40.5	20
fenvalerate-2		225.1	Wide	91	Wide	36.82	0.30	0.30	40.5	26
PBDE-154		645.6	Wide	485.7	Wide	37.61	0.30	0.30	84.8	26
PBDE-154		645.6	Wide	483.7	Wide	37.61	0.30	0.30	84.8	26
PBDE-154		643.6	Wide	483.7	Wide	37.61	0.30	0.30	84.8	26
PBDE-183		723.4	Wide	563.6	Wide	40.88	0.30	0.30	84.8	30
PBDE-183		723.4	Wide	561.6	Wide	40.88	0.30	0.30	84.8	30
PBDE-183		721.4	Wide	561.6	Wide	40.88	0.30	0.30	84.8	30
