# Supplementary material for: An exposome atlas of serum reveals the risk of chronic diseases in the Chinese population
Source: Nat Commun. 2024 Mar 13;15:2268. doi: 10.1038/s41467-024-46595-z (PMC10937660; doi:10.1038/s41467-024-46595-z)
Supplement: Supplementary file 6 — Reporting Summary [file 41467_2024_46595_MOESM6_ESM.pdf]

nature portfolio

Corresponding author(s): Guowang Xu

Last updated by author(s): Feb 18, 2024

Reporting Summary

Nature Portfolio wishes to improve the reproducibility of the work that we publish. This form provides structure for consistency and transparency in reporting. For further information on Nature Portfolio policies, see our Editorial Policies and the Editorial Policy Checklist.

Statistics

For all statistical analyses, confirm that the following items are present in the figure legend, table legend, main text, or Methods section.

n/a	Confirmed	
☐	☒	The exact sample size (*n*) for each experimental group/condition, given as a discrete number and unit of measurement
☐	☒	A statement on whether measurements were taken from distinct samples or whether the same sample was measured repeatedly
☐	☒	The statistical test(s) used AND whether they are one- or two-sided<br>*Only common tests should be described solely by name; describe more complex techniques in the Methods section.*
☐	☒	A description of all covariates tested
☐	☒	A description of any assumptions or corrections, such as tests of normality and adjustment for multiple comparisons
☐	☒	A full description of the statistical parameters including central tendency (e.g. means) or other basic estimates (e.g. regression coefficient) AND variation (e.g. standard deviation) or associated estimates of uncertainty (e.g. confidence intervals)
☐	☒	For null hypothesis testing, the test statistic (e.g. *F*, *t*, *r*) with confidence intervals, effect sizes, degrees of freedom and *P* value noted<br>*Give P values as exact values whenever suitable.*
☐	☒	For Bayesian analysis, information on the choice of priors and Markov chain Monte Carlo settings
☐	☒	For hierarchical and complex designs, identification of the appropriate level for tests and full reporting of outcomes
☐	☒	Estimates of effect sizes (e.g. Cohen's *d*, Pearson's *r*), indicating how they were calculated

Our web collection on statistics for biologists contains articles on many of the points above.

Software and code

Policy information about availability of computer code

Data collection	No code or software was used to collect the data.
Data analysis	No custom code was used in this study. The R codes for statistical analysis and figure production have been deposited to the GitHub (see https://github.com/youlei2023/ExposomeAtlas). The main data analysis packages include “ggplot2”, “vegan”, “FactoMineR”, “MatchIt”, “RSC”, “WQS”, “q g-comp” and “BKMR”.

For manuscripts utilizing custom algorithms or software that are central to the research but not yet described in published literature, software must be made available to editors and reviewers. We strongly encourage code deposition in a community repository (e.g. GitHub). See the Nature Portfolio guidelines for submitting code & software for further information.

Data

Policy information about availability of data

All manuscripts must include a data availability statement. This statement should provide the following information, where applicable:

- Accession codes, unique identifiers, or web links for publicly available datasets
- A description of any restrictions on data availability
- For clinical datasets or third party data, please ensure that the statement adheres to our policy

All data supporting the findings of this study are available within the paper, in the supplementary information file, and in the source data file. The air pollution dataset of China can be found in https://quotsoft.net/air/, and the surface climate dataset of China can be found in https://www.geodoi.ac.cn/WebCn/doi.aspx?

Id=3187. The concentration levels of 74 high-frequency exposures in human serum of Chinese chronic diseases population have been given in Table 2 of the paper, but the generated individual exposure atlas data are considered sensitive biomonitoring data, therefore, can not be publicly available according to the contracts with cooperating institutions (the initiator of the cohort) and the limitations included in the informed consents signed by the study participants. The request of these individual data is suggested by sending an email to the corresponding author Dr. Guowang Xu (xugw@dicp.ac.cn). Requests should include name, affiliation and contact details of the person requesting the data, which data are requested and the purpose of requesting the data. Requests will be subject to consideration by the management committee of the corresponding institutes and the sample collection institutes, including Dalian Institute of Chemical Physics, Chinese Academy of Sciences, National Institute for Nutrition and Health, Chinese Center for Disease Control and Prevention, Huazhong University of Science and Technology. If approved, the corresponding author will send the request data by email. Time frame for a response will be within 3 months. Data requests under agreement will be considered for purposes of reproducing the data and subject to appropriate confidentiality obligations and restrictions. Applicants must promise that these individual data applied for will only be used for scientific research and cannot be publicly released.

Human research participants

Policy information about studies involving human research participants and Sex and Gender in Research.

Reporting on sex and gender

Total of 2607males and 3089 females have been collected, the sex of participants was determined based on self-report, and the consent has been obtained. Findings in this study apply to both gender, and a part of gender-based analysis have also been performed. Detail results can been seen in the article.

Population characteristics

9 population characteristics have been included and adjusted as covariate in this study, they are age, gender, region, education level, income, sampling time, marital status, smoking and drinking history. The study population was from 15 provinces in China, with age ranging from 4 to 95 years (mean 51 ± 17 years). The ratio of male to female was 2607/3089. The average annual income was 22,211 ± 37,160 RMB. The proportion of smokers and non-smokers was 1396/4300. The proportion of drinking and non-drinking population was 1564/4132. In addition, the population included different levels of education, and different marital status. The detail covariate-relevant population characteristics were presented in Table 1 and Supplementary Figure 6.

Recruitment

A cross-sectional study containing 5696 subjects including 2141 healthy persons and 3555 patients was conducted in 15 provinces of China. The study population for this research is a subset of the cohort recruited from the China Nutrition and Health Survey in 2015. The cohort was selected using a multi-stage random cluster design, including 360 communities from 15 provinces with varying income levels. Then, 20 families were randomly selected from each community to participate in the study. Well-trained researchers collected epidemiological data and blood samples, The epidemiological factors were gathered by questionnaire, including gender, age, sampling location, sampling time, education level, income level, marital status, cigarette smoking, alcohol drinking and medical histories. And clinical parameters related to chronic diseases were determined through biochemical assays of the blood samples. For the case-control analysis, we strictly matched for sex, age, region, education level, income, sampling time, marital status, smoking, drinking history and other co-morbidities. Thus, the bias caused by these factors was removed. This information can be seen in method section in the main text.

Ethics oversight

The study has been approved by the National Institute for Nutrition and Health, Chinese Center for Disease Control and Prevention (reference No. 201524), and The name of the ethics committee is Chinese center for disease control and prevention institutional review board. Moreover a written informed consent was obtained from each participant before the study began.

Note that full information on the approval of the study protocol must also be provided in the manuscript.

Field-specific reporting

Please select the one below that is the best fit for your research. If you are not sure, read the appropriate sections before making your selection.

☐ Life sciences ☐ Behavioural & social sciences ☒ Ecological, evolutionary & environmental sciences

For a reference copy of the document with all sections, see nature.com/documents/nr-reporting-summary-flat.pdf

Ecological, evolutionary & environmental sciences study design

All studies must disclose on these points even when the disclosure is negative.

Study description

We designed a cohort of 5696 cases of normal and chronic disease patients with 12 chronic diseases, collecting 9 basic epidemiological factors, 9 clinical parameters of chronic disease and individual serum samples. Then, the human serum exposome including 267 chemicals was comprehensively determined by both GC-MS/MS and LC-MS/MS platforms, high-frequency chemicals have been further studied as the key targets of concern.

When the treatment factors were epidemiological factors, this is a hierarchical study design, all the participants (n=5696) were used and then grouped using basic epidemiological factors. Finally, the residue levels of chemicals in the serum were depicted in the stratified population to find out the exposure characteristics of people indifferent region, age, gender and etc.

When the treatment factors were disease parameters, this is a cross sectional case-control study. Case (chronic disease) and control (health) participants were selected using disease outcomes and propensity score matching. Associations between exposures and risk of chronic disease were established in the selected population using single exposure and mixture effect models together to specify

	chemical residues at risk for chronic diseases.
Research sample	Samples in this research were obtained from Chinese Center for Disease Control and Prevention. This cohort including 5696 cases of normal (n=2141) and chronic disease patients (n=3555). We chose natural populations from 15 provinces in China, and the rationale was multi-stage random cluster sampling. It is involved 360 communities, each community has 20 families according to income levels of different regions. In summary, these samples represent large natural populations in China. People from different regions, income levels, genders, ages and lifestyles were included in this cohort.
Sampling strategy	Sample size was defined according to the reference (doi: 10.1136/bmj.m441). For binary outcome, a sample size of at least 385 people is needed to target 95% confidence interval. The sample size of this study was 5696, therefore, it was adequate enough compare with the literature.
Data collection	Basic epidemiological factors of the cohort population were recorded by well trained interviewers using standard questionnaires. they are age, gender, region, education level, income, sampling time, marital status, smoking and drinking history.
Timing and spatial scale	The whole time period of sampling took place from August 1, 2015 to November 10, 2015. In the spatial scale, sampling was conducted in a manner that followed one province after another. The reason for this is to increase the sampling efficiency and to make the sampling time more concentrated, thus reducing the effect of differences caused by sampling time on the results. Samples from 4 to 5 provinces can be collected within one month, covering a total of 15 provinces in China, including northern, southern, coastal and inland regions.
Data exclusions	No data were excluded.
Reproducibility	In order to prove the repeatability of the experiment, we used quality control samples for monitoring during the sample detection process, inserting a quality control sample after every run of 11 real samples, and the quality control sample was repeatable, thus proving the repeatability of the sample analysis.
Randomization	In the risk analysis, chronic disease and healthy individuals were divided into case and control groups. To adjust confounders, propensity score matching was used to match control samples for each disease, for which control group was matched based on all nine epidemiological factors (age, gender, region, education level, income, sampling time, marital status, smoking and drinking history) and five major chronic diseases (obesity, hypertension, diabetes, hyperuricemia, and hyperlipidemia).
Blinding	This study is observational, and thus is not applicable for a blinding design.

Did the study involve field work? ☐ Yes ☒ No

Reporting for specific materials, systems and methods

We require information from authors about some types of materials, experimental systems and methods used in many studies. Here, indicate whether each material, system or method listed is relevant to your study. If you are not sure if a list item applies to your research, read the appropriate section before selecting a response.

Materials & experimental systems

n/a	Involved in the study
☒	☐ Antibodies
☒	☐ Eukaryotic cell lines
☒	☐ Palaeontology and archaeology
☒	☐ Animals and other organisms
☐	☒ Clinical data
☒	☐ Dual use research of concern

Methods

n/a	Involved in the study
☒	☐ ChIP-seq
☒	☐ Flow cytometry
☒	☐ MRI-based neuroimaging

Clinical data

Policy information about clinical studies

All manuscripts should comply with the ICMJE guidelines for publication of clinical research and a completed CONSORT checklist must be included with all submissions.

Clinical trial registration	Not Applicable.This study is based on epidemiological investigation cohort and does not involve any clinical intervention.
Study protocol	The study population in this work is a subset of the cohort recruited from the China Nutrition and Health Survey in 2015 which were described in previous studies. Therefore, the study protocol is available from the previous studies (doi: 10.1111/obr.12119. and doi: 10.3390/nu12061597.).
Data collection	Samples and patient characteristics were collected from August 1, 2015 to November 10, 2015 covering a total of 15 provinces in China, including northern, southern, coastal and inland regions.
Outcomes	The primary outcome was to determine whether there were significant differences in serum chemical residue concentrations

Outcomes

between the chronic disease patient and the health population, focusing on chemicals that significantly accumulated in the chronic disease patient, as well as chemical contaminants not reported in previous studies. Serum chemical residues were determined using both GC-MS/MS and LC-MS/MS methods. Chronic disease outcomes were determined using disease-related clinical parameters measured by serum biochemical experiments. The secondary outcome was to determine the levels of chemical residues in serum of the population by age, gender, region, education level, income, sampling time, marital status, smoking and drinking history. This demographic and lifestyle information was obtained using a questionnaire.
